# Supplementary material for: Choosing an Optimal Sample Preparation in Caulobacter crescentus for Untargeted Metabolomics Approaches
Source: Metabolites. 2019 Sep 20;9(10):193. doi: 10.3390/metabo9100193 (PMC6836107; doi:10.3390/metabo9100193)
Supplement: Supplementary file 1 [file metabolites-09-00193-s001.zip › Supp Data/Table S1.pdf]

Compound	m/z	Rt (min)	CCS (Å^2)	QC 1	QC 2	QC 3	QC 4	QC 5	QC 6	Mean	SD	RSD
1.34_581.2430m/z	581.2430	1.34	233.0	174.91	173.89	192.25	125.90	187.00	174.71	171.44	21.52	12.5%
7.28_260.0440m/z	260.0440	7.28	153.1	190.88	192.13	194.40	189.64	190.58	191.48	191.52	1.50	0.8%
9.44_606.0738m/z	606.0738	9.44	225.2	626.39	656.34	630.33	497.48	704.14	624.18	623.14	62.61	10.0%
7.12_266.0886m/z	266.0886	7.12	156.2	46.10	48.12	44.06	43.36	48.68	46.05	46.06	1.93	4.2%
8.58_288.1197m/z	288.1197	8.58	160.4	1363.14	1426.29	1324.65	1364.37	1357.55	1365.14	1366.86	30.06	2.2%
2.90_241.0722m/z	241.0722	2.90	135.9	2.16	1.92	0.00	2.92	1.80	2.16	1.83	0.89	48.8%
7.98_395.0540m/z	395.0540	7.98	177.6	0.00	0.00	0.00	0.00	0.00	0.00	0.00	0.00	141.4%
1.67_281.2484m/z	281.2484	1.67	179.4	16940.78	16842.31	16692.59	17439.09	16655.58	16939.94	16918.38	257.53	1.5%
8.48_165.0395m/z	165.0395	8.48	125.6	45.61	47.67	45.13	44.29	45.92	45.67	45.71	1.02	2.2%
1.28_281.2475m/z	281.2475	1.28	293.1	6.26	0.00	6.61	7.46	4.06	6.26	5.11	2.50	49.0%
0.96_347.2240m/z	347.2240	0.96	189.4	0.00	0.00	0.00	0.00	0.00	0.00	0.00	0.00	
8.04_349.0387m/z	349.0387	8.04	177.1	50.41	47.21	53.41	49.72	49.76	50.41	50.15	1.81	3.6%
9.59_606.0761m/z	606.0761	9.59	227.0	0.00	0.00	0.00	0.00	0.00	0.00	0.00	0.00	
1.31_281.2483m/z	281.2483	1.31	177.7	7366.69	7222.18	7584.17	7309.17	7258.44	7365.36	7351.00	116.71	1.6%
1.28_253.2164m/z	253.2164	1.28	170.2	2026.68	1933.39	2123.09	1991.77	2021.82	2025.30	2020.34	56.28	2.8%
8.48_808.1185m/z	808.1185	8.48	254.2	906.96	1105.74	999.11	1067.43	1007.34	1042.52	1021.52	62.54	6.1%
10.12_149.0088m/z	149.0088	10.12	118.9	39.18	34.07	41.52	40.27	42.02	39.07	39.36	2.60	6.6%
9.64_179.0557m/z	179.0557	9.64	131.0	29.51	29.02	30.69	27.39	31.01	29.46	29.51	1.18	4.0%
1.20_691.4193m/z	691.4193	1.20	267.4	0.00	6.34	6.56	6.81	0.00	6.48	4.36	3.09	70.8%
9.36_342.1162n	387.1144	9.36	176.1	13344.26	11683.17	13711.36	14337.42	12286.25	13347.91	13118.39	884.46	6.7%
8.67_130.0508m/z	130.0508	8.67	125.3	142.31	140.45	144.85	144.97	136.80	142.38	141.96	2.79	2.0%
1.56_482.2943m/z	482.2943	1.56	209.2	160022.03	157694.76	159233.66	163187.60	158795.33	159967.96	159816.89	1698.71	1.1%
8.90_342.1161n	341.1088	8.90	168.8	1153.34	1120.48	1137.64	1191.08	1154.79	1152.58	1151.65	21.35	1.9%
9.46_711.2175m/z	711.2175	9.46	247.9	2.63	2.52	0.00	2.98	0.00	2.64	1.79	1.28	71.2%
10.62_189.0880m/z	189.0880	10.62	136.9	38.86	42.37	11.12	63.95	44.81	38.78	39.98	15.49	38.7%
8.20_163.0606m/z	163.0606	8.20	128.9	143.29	146.54	142.73	142.08	142.08	143.35	143.35	1.52	1.1%
9.29_549.1679m/z	549.1679	9.29	220.6	0.00	0.00	0.00	0.00	0.00	0.00	0.00	0.00	
9.79_225.0987m/z	225.0987	9.79	149.6	8.65	8.80	6.52	9.48	8.86	8.64	8.49	0.93	10.9%

10.57_408.0130m/z	408.0130	10.57	172.2	21.07	23.09	7.45	22.97	23.99	21.04	19.93	5.69	28.5%
9.39_214.0480m/z	214.0480	9.39	142.0	282.14	252.61	302.00	287.85	264.60	282.06	278.54	15.96	5.7%
8.37_290.0877m/z	290.0877	8.37	158.6	498.00	511.65	498.08	488.42	498.74	498.43	498.89	6.76	1.4%
11.12_220.9626m/z	220.9626	11.12	143.3	0.00	0.00	0.02	0.12	0.11	0.00	0.04	0.05	127.3%
8.64_202.0717m/z	202.0717	8.64	142.6	209.42	207.18	213.68	208.26	206.63	209.40	209.09	2.30	1.1%
9.91_866.1197m/z	866.1197	9.91	253.5	2.71	2.06	0.00	1.95	3.51	2.52	2.13	1.08	50.7%
9.78_565.0474m/z	565.0474	9.78	213.1	272.62	242.41	270.33	289.81	287.54	271.86	272.43	15.47	5.7%
8.65_383.1135m/z	383.1135	8.65	186.6	152.98	153.80	154.27	154.52	148.25	153.09	152.82	2.12	1.4%
1.93_405.1906m/z	405.1906	1.93	202.0	0.00	0.00	0.00	0.00	0.00	0.00	0.00	0.00	
2.54_391.2868m/z	391.2868	2.54	211.3	1.63	1.36	0.00	0.00	2.16	1.17	1.06	0.81	76.3%
2.28_407.2797m/z	407.2797	2.28	205.5	20256.04	20382.49	18804.57	21704.13	19725.33	20259.19	20188.63	863.53	4.3%
7.01_266.0887m/z	266.0887	7.01	159.5	4.68	1.84	2.06	6.22	3.14	4.69	3.77	1.57	41.6%
11.08_338.9880m/z	338.9880	11.08	152.2	10.04	10.26	9.68	0.00	0.00	10.01	6.67	4.72	70.8%
10.28_426.0207m/z	426.0207	10.28	182.1	76.89	83.82	63.17	77.79	78.86	76.91	76.24	6.30	8.3%
8.25_299.0998m/z	299.0998	8.25	161.7	172.46	174.75	177.05	164.89	172.93	172.52	172.43	3.74	2.2%
8.84_307.0826n	306.0764	8.84	161.4	14301.27	15859.70	13249.99	14472.12	14555.70	14481.94	14486.79	758.72	5.2%
9.13_445.0526m/z	445.0526	9.13	178.2	0.00	0.00	0.00	0.00	0.00	0.00	0.00	0.00	
8.30_171.0406m/z	171.0406	8.30	131.6	140.68	139.12	143.70	138.06	141.24	140.61	140.57	1.76	1.3%
9.80_193.0352m/z	193.0352	9.80	131.8	0.48	0.00	0.47	0.53	0.48	0.48	0.41	0.18	45.0%
6.88_193.0727m/z	193.0727	6.88	139.9	1025.08	1006.40	1033.33	1028.98	1025.95	1024.51	1024.04	8.44	0.8%
9.03_187.1084m/z	187.1084	9.03	145.1	0.00	7.18	7.36	7.16	7.39	7.27	6.06	2.71	44.7%
9.08_160.0614m/z	160.0614	9.08	129.1	49.30	50.54	50.21	45.45	50.72	49.29	49.25	1.79	3.6%
8.94_766.1078m/z	766.1078	8.94	249.0	2.16	776.99	716.64	785.60	823.73	781.54	647.78	290.43	44.8%
10.14_341.1074m/z	341.1074	10.14	170.5	26.93	25.30	24.16	32.72	24.06	26.95	26.69	2.94	11.0%
8.57_180.0630n	179.0561	8.57	131.0	7950.07	8028.21	7996.43	7847.79	7938.13	7950.38	7951.84	56.04	0.7%
9.29_211.0002m/z	211.0002	9.29	134.1	0.00	0.00	0.00	0.00	0.00	0.00	0.00	0.00	
6.71_162.0418m/z	162.0418	6.71	125.8	347.90	344.43	350.07	345.61	350.80	347.69	347.75	2.25	0.6%
6.12_134.0463m/z	134.0463	6.12	170.3	0.00	0.00	0.00	0.00	0.00	0.00	0.00	0.00	141.8%
9.02_188.0563m/z	188.0563	9.02	136.9	1324.02	1467.60	1415.75	1344.48	1398.12	1402.88	1392.14	47.11	3.4%

8.96_369.0099m/z	369.0099	8.96	168.1	32.74	28.87	39.89	1.74	0.00	12.95	19.37	15.37	79.4%
9.34_322.0443m/z	322.0443	9.34	162.6	486.96	485.14	490.71	466.57	507.04	486.31	487.12	11.81	2.4%
5.75_156.0661m/z	156.0661	5.75	134.3	9.11	7.02	10.89	8.16	10.01	9.07	9.04	1.24	13.7%
7.65_128.0346m/z	128.0346	7.65	178.3	20.02	17.69	20.92	19.85	21.07	18.16	19.62	1.28	6.5%
8.58_383.0600m/z	383.0600	8.58	176.2	11.34	12.26	9.70	13.58	6.15	11.42	10.74	2.36	22.0%
10.91_505.9879m/z	505.9879	10.91	189.2	13.93	15.51	9.81	0.00	8.96	13.94	10.36	5.19	50.1%
8.24_283.0673m/z	283.0673	8.24	158.9	97.88	99.01	97.26	97.39	98.18	97.87	97.93	0.57	0.6%
5.99_312.0946m/z	312.0946	5.99	164.6	2295.21	2248.43	2371.14	2219.43	2325.64	2293.54	2292.23	49.28	2.1%
8.81_202.0714m/z	202.0714	8.81	139.4	5.26	34.95	24.55	31.25	30.14	30.23	26.06	9.79	37.6%
8.85_540.0537m/z	540.0537	8.85	213.5	4982.45	5543.37	4489.05	5033.41	5097.38	5025.36	5028.50	306.32	6.1%
8.98_147.0527n	146.0457	8.98	123.9	905.38	892.03	954.55	866.43	893.01	905.38	902.80	26.54	2.9%
1.90_391.2842m/z	391.2842	1.90	204.1	4330.42	4252.60	4338.24	4391.26	4302.84	4328.90	4324.04	41.51	1.0%
8.47_344.0398m/z	344.0398	8.47	170.4	119.31	123.32	120.54	110.58	123.95	119.31	119.50	4.38	3.7%
2.17_163.0407m/z	163.0407	2.17	135.3	0.00	0.00	0.00	0.00	0.00	0.00	0.00	0.00	
11.17_579.0267m/z	579.0267	11.17	212.8	2640.43	2677.59	2792.19	2046.45	2643.92	2640.69	2573.54	241.66	9.4%
8.25_330.0604m/z	330.0604	8.25	170.8	132.24	133.53	133.79	131.01	130.50	132.31	132.23	1.20	0.9%
5.14_448.3063m/z	448.3063	5.14	200.9	14597.28	13675.35	15611.84	13898.55	14954.85	14576.76	14552.44	643.39	4.4%
1.13_227.2020m/z	227.2020	1.13	166.3	0.00	0.00	0.00	0.00	0.00	0.00	0.00	0.00	223.6%
8.92_195.0506m/z	195.0506	8.92	131.7	721.15	791.82	661.48	716.76	746.43	725.80	727.24	38.75	5.3%
5.79_173.0816m/z	173.0816	5.79	134.6	483.96	559.89	351.90	542.87	459.75	487.17	480.92	67.35	14.0%
5.72_465.3091n	464.3018	5.72	204.2	190587.71	186927.09	193350.38	190110.93	190811.33	190483.99	190378.57	1873.42	1.0%
2.62_391.2872m/z	391.2872	2.62	207.7	9.57	8.14	6.37	11.93	7.95	9.59	8.92	1.73	19.4%
8.21_321.0492m/z	321.0492	8.21	162.6	674.51	686.50	670.70	673.61	669.38	674.74	674.91	5.54	0.8%
10.71_442.0167m/z	442.0167	10.71	181.8	125.11	136.17	66.38	179.62	148.40	124.90	130.10	34.00	26.1%
8.68_219.0508m/z	219.0508	8.68	141.7	0.00	1.21	0.00	1.37	1.11	1.22	0.82	0.58	71.3%
9.19_347.0403m/z	347.0403	9.19	170.3	69.57	72.26	67.52	70.84	68.12	69.62	69.66	1.59	2.3%
9.34_377.0850m/z	377.0850	9.34	172.9	304.89	254.91	343.88	242.23	340.75	302.98	298.27	38.67	13.0%
7.65_128.0351m/z	128.0351	7.65	123.9	559.34	552.21	572.63	540.50	571.71	558.77	559.19	11.07	2.0%
8.73_177.0396m/z	177.0396	8.73	128.0	0.00	1.19	1.35	0.17	3.42	1.19	1.22	1.11	91.4%

9.47_427.0068m/z	427.0068	9.47	176.9	2.18	371.44	481.36	32.62	458.10	365.90	285.27	194.17	68.1%
11.78_521.9850m/z	521.9850	11.78	195.9	4.20	4.15	4.39	4.04	4.18	4.20	4.20	0.10	2.5%
9.43_362.0497m/z	362.0497	9.43	169.9	263.22	253.95	269.64	248.56	277.44	262.67	262.58	9.51	3.6%
7.84_282.0840m/z	282.0840	7.84	158.9	1951.56	1911.71	1971.97	1968.32	1937.64	1950.81	1948.67	20.12	1.0%
2.16_164.0347m/z	164.0347	2.16	132.0	0.00	0.00	0.00	0.00	0.00	0.00	0.00	0.00	
6.43_218.1028m/z	218.1028	6.43	146.7	242.80	252.10	235.72	237.44	246.69	242.79	242.92	5.48	2.3%
8.71_237.0613m/z	237.0613	8.71	145.8	161.46	178.33	157.71	150.21	172.76	164.07	164.09	9.29	5.7%
7.30_130.0872m/z	130.0872	7.30	130.1	1369.54	1354.47	1402.07	1314.71	1404.94	1368.21	1368.99	30.40	2.2%
7.60_203.0825m/z	203.0825	7.60	149.1	2920.56	2879.57	2953.20	2916.56	2917.08	2919.29	2917.71	21.32	0.7%
9.13_154.0622m/z	154.0622	9.13	128.0	57.11	41.88	69.38	62.91	54.49	57.08	57.14	8.40	14.7%
9.34_131.0459m/z	131.0459	9.34	197.9	11.21	0.00	18.50	17.39	16.34	17.37	13.47	6.46	48.0%
11.22_481.9749m/z	481.9749	11.22	182.7	8.54	8.77	9.52	1.14	7.69	8.55	7.37	2.84	38.5%
8.16_242.0778m/z	242.0778	8.16	153.8	399.16	402.02	400.72	394.30	399.80	399.10	399.18	2.40	0.6%
7.09_164.0717m/z	164.0717	7.09	138.5	6193.97	6100.05	6210.53	6205.54	6239.68	6189.78	6189.92	43.28	0.7%
10.38_402.0097m/z	402.0097	10.38	172.3	1.00	1.01	0.00	0.00	9.59	0.96	2.09	3.38	161.6%
8.56_347.0631n	346.0558	8.56	172.1	9061.36	9428.95	8880.69	9041.16	9026.15	9074.74	9085.51	166.38	1.8%
7.14_328.0448m/z	328.0448	7.14	167.5	230.70	231.29	226.05	230.14	236.05	230.47	230.78	2.91	1.3%
8.27_180.0665m/z	180.0665	8.27	142.3	7073.02	6963.12	7191.70	7002.48	7101.21	7069.04	7066.76	72.70	1.0%
8.97_323.0285m/z	323.0285	8.97	160.9	3390.36	3757.76	3059.03	3355.57	3482.70	3407.13	3408.76	205.24	6.0%
6.33_243.0622m/z	243.0622	6.33	148.8	4058.35	4002.61	4126.76	4033.30	4044.27	4056.88	4053.69	37.60	0.9%
9.64_426.0218m/z	426.0218	9.64	183.8	745.68	739.75	716.43	707.46	805.47	743.33	743.02	31.33	4.2%
7.01_267.0731m/z	267.0731	7.01	156.1	1507.39	1508.90	1478.86	1544.54	1491.09	1507.19	1506.33	20.23	1.3%
8.90_387.1141m/z	387.1141	8.90	174.4	394.01	372.55	408.78	387.82	404.63	393.49	393.55	11.75	3.0%
9.19_145.0615m/z	145.0615	9.19	130.3	0.00	0.00	0.00	0.00	0.00	0.00	0.00	0.00	
9.61_259.0217m/z	259.0217	9.61	144.9	206.09	210.76	195.49	202.58	215.89	205.79	206.10	6.36	3.1%
10.27_505.9880m/z	505.9880	10.27	189.2	218.62	214.45	101.64	302.58	249.04	217.85	217.36	60.09	27.6%
10.31_744.0846m/z	744.0846	10.31	226.9	0.00	0.00	0.00	0.00	0.00	0.00	0.00	0.00	141.5%
8.67_176.0562m/z	176.0562	8.67	129.6	13.22	14.30	12.27	12.45	14.27	13.23	13.29	0.79	5.9%
1.44_691.4191m/z	691.4191	1.44	269.3	0.00	34.29	37.78	29.96	37.39	34.89	29.05	13.24	45.6%

0.73_283.2640m/z	283.2640	0.73	181.1	332.80	320.27	354.75	309.78	340.37	332.42	331.73	14.25	4.3%
2.40_201.1139m/z	201.1139	2.40	145.9	0.00	0.00	0.00	0.00	0.00	0.00	0.00	0.00	223.6%
2.17_107.0498m/z	107.0498	2.17	120.0	8.62	8.59	0.00	8.97	9.08	8.52	7.30	3.27	44.8%
9.03_171.0061m/z	171.0061	9.03	125.2	92.87	104.58	81.87	92.97	95.70	93.69	93.61	6.63	7.1%
4.15_187.0972m/z	187.0972	4.15	140.2	4837.75	4822.22	4686.28	4938.70	4873.68	4835.07	4832.29	75.82	1.6%
5.88_245.0930m/z	245.0930	5.88	157.0	49.40	49.13	48.87	49.06	50.45	49.36	49.38	0.51	1.0%
7.80_161.0450m/z	161.0450	7.80	127.5	123.79	122.06	126.56	117.23	129.66	123.56	123.81	3.84	3.1%
6.67_151.0262m/z	151.0262	6.67	120.3	7431.72	7316.82	7443.40	7537.54	7377.29	7429.09	7422.64	67.10	0.9%
8.45_167.0210m/z	167.0210	8.45	123.9	1002.00	1020.78	986.64	1010.57	993.30	1002.31	1002.60	11.07	1.1%
2.26_212.0198m/z	212.0198	2.26	148.6	0.00	0.00	0.00	0.00	0.00	0.00	0.00	0.00	
6.12_218.1031m/z	218.1031	6.12	146.7	1212.94	1200.60	1232.33	1188.04	1228.08	1212.14	1212.36	15.14	1.2%
1.66_255.2323m/z	255.2323	1.66	173.6	7917.23	7796.53	7886.90	8082.47	7832.77	7915.20	7905.19	90.43	1.1%
6.38_135.0313m/z	135.0313	6.38	118.5	2117.18	2067.55	2154.12	2110.73	2121.16	2115.90	2114.44	25.31	1.2%
7.49_150.0415m/z	150.0415	7.49	123.5	129.09	126.12	129.41	126.17	137.20	122.48	128.41	4.55	3.5%
8.46_177.0403m/z	177.0403	8.46	131.2	22.89	24.81	20.18	27.06	21.88	23.92	23.46	2.18	9.3%
5.67_295.1384m/z	295.1384	5.67	166.9	59.18	57.64	59.66	38.54	65.97	27.79	51.46	13.56	26.3%
10.07_191.0195m/z	191.0195	10.07	125.6	227.55	221.15	157.46	274.75	255.26	226.73	227.15	36.40	16.0%
5.90_134.0472m/z	134.0472	5.90	118.6	4174.69	4061.37	4285.83	4129.24	4185.61	4172.11	4168.14	67.24	1.6%
1.56_121.0293m/z	121.0293	1.56	118.3	19.22	0.00	20.19	19.82	19.75	19.69	16.44	7.36	44.8%
8.83_306.2357m/z	306.2357	8.83	161.4	90.45	232.57	190.39	203.51	213.05	208.91	189.81	46.18	24.3%
8.83_361.1353m/z	361.1353	8.83	176.8	344.02	473.82	383.62	419.59	431.18	425.32	412.93	40.51	9.8%
8.83_164.0712m/z	164.0712	8.83	138.5	69.84	76.12	69.03	68.52	72.84	71.23	71.26	2.60	3.7%
8.79_413.1679m/z	413.1679	8.79	191.1	2427.61	2697.24	2272.70	2425.74	2498.25	2463.33	2464.15	125.80	5.1%
8.79_683.3011m/z	683.3011	8.79	248.3	0.00	78.48	78.29	81.86	78.15	79.35	66.02	29.55	44.8%
8.82_655.2248m/z	655.2248	8.82	224.4	0.00	23.89	19.58	27.45	23.42	24.04	19.73	9.11	46.2%
8.81_442.1914m/z	442.1914	8.81	201.1	126.41	146.69	124.03	137.76	131.90	134.63	133.57	7.48	5.6%
8.82_504.2041m/z	504.2041	8.82	165.3	123.55	141.63	132.12	129.62	130.81	133.40	131.85	5.37	4.1%
8.82_524.2460m/z	524.2460	8.82	217.4	755.90	797.04	735.76	749.42	760.01	757.92	759.34	18.67	2.5%
8.80_624.2656m/z	624.2656	8.80	236.0	93.18	177.47	149.73	157.70	160.50	160.59	149.86	26.66	17.8%

8.81_444.2291m/z	444.2291	8.81	166.4	631.89	653.70	668.16	582.70	661.18	640.68	639.72	28.22	4.4%
8.91_428.1773m/z	428.1773	8.91	196.1	257.82	304.55	260.78	269.90	283.24	278.23	275.75	15.66	5.7%
8.94_551.2456m/z	551.2456	8.94	226.1	1528.96	1668.95	1422.64	1554.79	1532.59	1539.74	1541.28	71.63	4.6%
8.93_468.6872m/z	468.6872	8.93	159.3	16.32	17.38	16.20	17.25	15.58	16.62	16.56	0.62	3.7%
8.78_255.0472m/z	255.0472	8.78	150.0	659.36	732.36	587.91	653.45	676.95	662.04	662.01	42.31	6.4%
8.85_370.5782m/z	370.5782	8.85	144.9	104.54	111.67	101.31	103.47	105.69	105.10	105.30	3.18	3.0%
8.85_541.2246m/z	541.2246	8.85	217.1	849.28	1030.07	844.48	923.21	934.19	929.62	918.48	62.06	6.8%
8.85_444.1731m/z	444.1731	8.85	193.9	1139.30	1373.90	1084.84	1226.49	1244.46	1228.70	1216.28	90.46	7.4%
8.85_259.0916m/z	259.0916	8.85	161.5	1595.84	1342.63	1714.89	1703.43	1552.50	1594.77	1584.01	123.09	7.8%
8.84_342.5655m/z	342.5655	8.84	142.4	93.54	100.28	86.88	95.18	93.38	93.73	93.83	3.92	4.2%
8.87_382.5492m/z	382.5492	8.87	143.0	2065.07	2232.93	1921.59	2083.49	2071.85	2071.69	2074.44	90.06	4.3%
8.89_459.6811m/z	459.6811	8.89	159.4	232.97	245.23	224.69	230.60	235.47	233.24	233.70	6.16	2.6%
8.89_306.0476m/z	306.0476	8.89	161.4	228.73	248.60	215.55	222.28	238.70	229.90	230.63	10.72	4.6%
8.88_302.1340m/z	302.1340	8.88	166.6	221.20	290.13	252.82	260.16	268.93	266.63	259.98	20.77	8.0%
8.87_383.0500m/z	383.0500	8.87	141.4	370.76	398.95	352.76	364.14	378.37	372.01	372.83	14.12	3.8%
8.87_284.1286m/z	284.1286	8.87	167.3	574.21	606.34	555.92	557.36	588.19	574.82	576.14	17.44	3.0%
8.87_245.1140m/z	245.1140	8.87	157.0	1651.22	2494.57	2085.09	2399.95	2179.03	2287.61	2182.91	273.09	12.5%
8.75_359.1554m/z	359.1554	8.75	182.0	115.15	244.08	206.10	215.40	225.42	221.51	204.61	41.63	20.3%
8.64_315.2027m/z	315.2027	8.64	156.1	716.16	729.25	705.41	726.16	704.35	716.48	716.30	9.37	1.3%
8.64_280.1016m/z	280.1016	8.64	157.3	79.30	80.71	79.57	78.84	78.66	79.39	79.41	0.66	0.8%
8.64_470.1874m/z	470.1874	8.64	204.0	327.02	346.34	319.53	331.79	320.84	329.15	329.11	8.84	2.7%
8.64_376.9654m/z	376.9654	8.64	159.5	350.58	375.43	346.90	349.57	353.45	355.15	355.18	9.44	2.7%
8.64_315.3085m/z	315.3085	8.64	156.1	173.11	178.14	166.09	180.37	167.33	173.26	173.05	5.17	3.0%
8.64_573.2379n	572.2321	8.64	222.1	1337.06	1437.05	1300.11	1331.47	1340.02	1347.89	1348.93	42.18	3.1%
8.63_315.5429m/z	315.5429	8.63	156.1	122.73	129.62	114.33	128.02	119.63	122.92	122.88	5.09	4.1%
8.64_316.0488n	315.0416	8.64	154.5	35051.88	35809.06	34350.80	35524.16	34620.95	35066.22	35070.51	495.26	1.4%
8.66_195.0506m/z	195.0506	8.66	133.3	250.02	255.91	247.97	250.70	248.53	250.37	250.58	2.58	1.0%
8.65_588.1086m/z	588.1086	8.65	227.3	26.67	27.81	26.33	27.44	26.17	26.91	26.89	0.58	2.2%
8.65_633.7862m/z	633.7862	8.65	185.5	213.24	228.55	203.03	217.28	213.99	214.84	215.16	7.48	3.5%

8.62_660.0431m/z	660.0431	8.62	220.7	0.00	374.21	381.88	369.81	388.82	378.31	315.50	141.22	44.8%
8.60_504.1757m/z	504.1757	8.60	212.4	264.87	275.67	262.25	261.04	264.11	265.30	265.54	4.76	1.8%
8.59_265.1116n	529.2250	8.59	211.9	258.20	275.55	247.81	259.10	257.06	259.22	259.49	8.19	3.2%
8.59_589.7485m/z	589.7485	8.59	180.9	80.19	79.11	78.24	84.36	77.44	80.22	79.93	2.22	2.8%
8.60_696.0196m/z	696.0196	8.60	227.5	0.00	115.87	126.76	98.34	126.82	117.53	97.55	44.65	45.8%
8.58_344.2738m/z	344.2738	8.58	157.0	22.97	29.81	23.18	26.62	27.11	26.58	26.04	2.37	9.1%
8.58_256.0146m/z	256.0146	8.58	151.6	21.79	24.05	17.07	25.32	18.88	21.88	21.50	2.82	13.1%
8.59_345.0015n	343.9948	8.59	155.3	16865.89	17067.40	16776.57	17038.94	16554.41	16872.89	16862.68	171.04	1.0%
8.57_653.2883m/z	653.2883	8.57	237.4	86.76	93.25	79.90	95.04	75.87	87.26	86.35	6.78	7.8%
8.60_429.1852m/z	429.1852	8.60	196.0	35.31	37.73	34.23	30.66	38.85	35.25	35.34	2.62	7.4%
8.62_551.0378m/z	551.0378	8.62	199.0	102.86	114.73	95.26	115.59	92.95	105.59	104.50	8.66	8.3%
8.61_808.3434m/z	808.3434	8.61	261.9	0.00	77.30	77.81	73.94	83.27	77.92	65.04	29.21	44.9%
8.61_454.1921m/z	454.1921	8.61	202.6	240.13	255.84	234.31	239.84	241.91	242.02	242.34	6.56	2.7%
8.61_341.9993m/z	341.9993	8.61	157.0	771.18	780.86	773.46	769.87	759.33	771.50	771.03	6.34	0.8%
8.60_344.1633m/z	344.1633	8.60	157.0	260.52	264.05	260.13	261.60	256.51	260.66	260.58	2.23	0.9%
8.60_669.2827m/z	669.2827	8.60	240.9	47.37	51.23	45.55	49.95	45.89	48.16	48.03	2.05	4.3%
8.62_257.1139m/z	257.1139	8.62	154.9	646.39	656.24	654.89	625.21	651.39	646.43	646.76	10.35	1.6%
8.74_356.1680n	355.1607	8.74	180.4	721.65	764.86	704.56	710.54	730.32	724.63	726.09	19.36	2.7%
8.73_513.2262m/z	513.2262	8.73	214.0	57.92	77.48	75.04	68.20	74.53	73.62	71.13	6.54	9.2%
8.72_371.1551m/z	371.1551	8.72	180.0	35.16	37.47	35.18	35.94	34.19	35.64	35.60	1.00	2.8%
8.72_399.1500m/z	399.1500	8.72	182.7	64.88	88.10	67.82	80.06	79.04	78.51	76.40	7.83	10.2%
8.72_418.1570m/z	418.1570	8.72	187.5	122.56	205.15	178.84	187.12	188.19	188.77	178.44	26.18	14.7%
8.72_598.2468m/z	598.2468	8.72	223.5	808.05	949.56	808.62	873.77	861.41	869.95	861.89	47.66	5.5%
8.75_339.9998m/z	339.9998	8.75	157.1	131.55	142.89	122.05	130.49	131.76	131.84	131.76	6.05	4.6%
8.77_556.2349m/z	556.2349	8.77	215.0	442.49	495.26	432.32	461.76	448.31	458.08	456.37	19.92	4.4%
8.77_456.1727m/z	456.1727	8.77	193.7	541.88	671.36	529.42	603.95	602.89	600.24	591.62	46.65	7.9%
8.77_628.2589m/z	628.2589	8.77	228.5	2665.63	3031.37	2563.34	2796.48	2765.01	2777.35	2766.53	142.96	5.2%
8.76_624.2582m/z	624.2582	8.76	236.0	25.66	41.96	40.85	40.53	37.76	40.44	37.87	5.60	14.8%
8.68_286.1033m/z	286.1033	8.68	157.1	234.68	257.92	227.90	229.58	244.04	238.68	238.80	10.11	4.2%

8.67_447.1524m/z	447.1524	8.67	197.4	52.71	61.57	49.56	58.36	55.82	56.11	55.69	3.84	6.9%
8.67_415.1715m/z	415.1715	8.67	191.1	0.00	8.32	7.40	8.41	8.75	8.32	6.87	3.10	45.1%
8.71_583.2410m/z	583.2410	8.71	221.9	0.00	11.98	9.76	13.89	11.64	12.03	9.88	4.58	46.3%
8.70_514.2157m/z	514.2157	8.70	210.4	72.04	102.29	80.18	96.39	89.51	91.96	88.73	10.04	11.3%
8.70_695.3004m/z	695.3004	8.70	248.1	568.83	626.65	536.07	582.52	579.85	578.56	578.75	26.55	4.6%
8.69_232.0932m/z	232.0932	8.69	149.3	103.90	107.15	104.88	100.27	105.21	104.11	104.25	2.07	2.0%
8.69_165.0401m/z	165.0401	8.69	125.6	214.92	222.02	216.86	209.83	216.77	215.78	216.03	3.58	1.7%
9.19_284.0318m/z	284.0318	9.19	114.4	2609.10	2671.71	2575.92	2568.87	2637.05	2608.86	2611.92	35.05	1.3%
9.24_478.0509n	513.0234	9.24	150.4	4933.21	5812.56	5231.51	4876.37	5060.39	5342.30	5209.39	313.94	6.0%
9.26_164.0111m/z	164.0111	9.26	110.2	33715.56	34524.39	33374.87	33176.25	33979.32	33715.76	33747.69	432.92	1.3%
9.25_349.0122n	348.0049	9.25	131.2	6724.83	7412.38	6594.40	6301.33	6497.84	6811.60	6723.73	348.36	5.2%
9.25_164.5125m/z	164.5125	9.25	110.2	3326.41	3370.64	3295.66	3342.83	3299.05	3326.86	3326.91	25.56	0.8%
9.01_276.0954n	231.0983	9.01	152.7	1093.94	1151.69	1131.20	1097.13	1100.75	1119.23	1115.66	20.78	1.9%
9.00_257.0776m/z	257.0776	9.00	158.2	812.95	930.83	792.26	813.76	864.75	847.04	843.60	45.71	5.4%
9.01_300.1212m/z	300.1212	9.01	166.7	0.00	1.74	1.91	1.22	2.01	1.75	1.44	0.69	48.0%
9.04_243.0178m/z	243.0178	9.04	140.7	1506.76	1639.62	1381.36	1471.80	1558.76	1509.35	1511.27	78.73	5.2%
9.05_215.0671m/z	215.0671	9.05	140.3	457.02	480.02	459.34	425.19	470.61	457.65	458.30	16.95	3.7%
8.54_242.0795m/z	242.0795	8.54	153.8	1074.25	1117.52	1066.17	1051.85	1077.71	1075.91	1077.24	20.00	1.9%
7.81_397.2072m/z	397.2072	7.81	191.5	2052.15	2022.04	2063.67	2093.94	2005.50	2052.10	2048.23	28.49	1.4%
7.81_412.1830m/z	412.1830	7.81	191.1	38.16	35.27	38.77	40.12	36.81	38.15	37.88	1.52	4.0%
7.81_341.1817m/z	341.1817	7.81	179.1	106.80	104.60	102.35	118.04	95.92	106.92	105.77	6.62	6.3%
7.81_462.1981m/z	462.1981	7.81	202.4	306.34	303.95	300.26	315.75	303.42	306.25	305.99	4.81	1.6%
7.80_334.1403m/z	334.1403	7.80	179.3	2398.93	2391.08	2390.64	2433.81	2367.13	2398.76	2396.73	19.70	0.8%
7.83_186.0632n	185.0565	7.83	140.4	856.96	838.16	872.29	839.18	875.57	856.03	856.36	14.43	1.7%
7.83_415.2122m/z	415.2122	7.83	203.5	9.58	9.71	9.20	10.38	8.62	9.59	9.51	0.53	5.6%
7.83_442.2127m/z	442.2127	7.83	199.3	21.87	21.15	23.77	20.24	21.32	21.87	21.70	1.07	4.9%
7.83_605.2558m/z	605.2558	7.83	228.9	201.06	197.35	201.30	202.37	202.67	200.87	200.94	1.74	0.9%
7.83_395.1563m/z	395.1563	7.83	189.8	839.46	830.83	846.53	830.32	849.28	838.86	839.21	7.12	0.8%
7.83_270.1454m/z	270.1454	7.83	164.4	598.52	584.84	607.88	601.36	594.36	598.28	597.54	7.00	1.2%

7.82_221.0925m/z	221.0925	7.82	151.5	199.01	195.13	200.75	199.59	199.53	198.88	198.82	1.75	0.9%
7.76_243.1349m/z	243.1349	7.76	158.8	4725.39	4697.71	4765.79	4665.84	4771.90	4722.49	4724.85	36.78	0.8%
7.76_371.1885m/z	371.1885	7.76	188.7	155.63	149.36	147.60	177.32	136.97	155.77	153.78	12.26	8.0%
7.76_703.3052m/z	703.3052	7.76	246.1	1181.59	1170.18	1167.88	1222.53	1152.57	1181.64	1179.40	21.63	1.8%
7.79_640.2936m/z	640.2936	7.79	235.8	222.33	215.05	227.81	214.76	232.19	221.71	222.31	6.30	2.8%
7.79_274.1397m/z	274.1397	7.79	164.2	205.15	201.02	205.81	199.60	215.64	204.51	205.29	5.14	2.5%
7.78_483.1887m/z	483.1887	7.78	196.7	7.85	6.89	8.91	6.52	8.60	7.71	7.75	0.85	11.0%
7.78_458.2251m/z	458.2251	7.78	204.3	998.47	980.58	1007.41	1003.83	995.38	998.03	997.28	8.47	0.8%
7.81_601.2697n	582.2537	7.81	223.7	1765.59	1748.66	1791.02	1706.01	1825.79	1762.84	1766.65	36.76	2.1%
7.95_667.3050m/z	667.3050	7.95	241.0	523.42	520.23	527.74	515.05	530.99	523.04	523.41	5.10	1.0%
7.94_315.1655m/z	315.1655	7.94	173.0	30.67	29.91	30.28	32.09	29.87	30.67	30.58	0.75	2.4%
7.93_965.4613m/z	965.4613	7.93	289.4	97.30	98.15	99.66	85.52	106.28	96.90	97.30	6.14	6.3%
7.93_567.2449m/z	567.2449	7.93	222.1	50.93	46.47	55.84	44.85	55.45	50.65	50.70	4.11	8.1%
7.93_185.0215m/z	185.0215	7.93	132.3	64.92	63.87	64.91	65.37	65.29	64.87	64.87	0.49	0.8%
7.93_371.1917m/z	371.1917	7.93	186.9	701.05	698.91	705.51	678.57	724.95	700.09	701.51	13.54	1.9%
7.93_227.0667m/z	227.0667	7.93	149.5	219.43	213.77	222.63	219.22	220.62	219.26	219.16	2.69	1.2%
7.96_391.1624m/z	391.1624	7.96	191.7	210.44	208.52	207.70	219.67	202.40	210.50	209.87	5.15	2.5%
7.99_273.1075m/z	273.1075	7.99	164.3	39.65	39.08	40.98	38.19	39.93	39.62	39.57	0.84	2.1%
7.99_426.1626m/z	426.1626	7.99	189.1	291.01	285.30	293.22	292.00	292.01	290.81	290.72	2.55	0.9%
7.98_543.2387m/z	543.2387	7.98	215.3	44.06	44.62	44.06	42.14	45.90	44.00	44.13	1.11	2.5%
7.98_784.3534m/z	784.3534	7.98	256.4	40.68	38.82	40.65	42.81	39.10	40.68	40.46	1.30	3.2%
7.98_678.2935m/z	678.2935	7.98	188.4	196.82	192.84	195.27	207.28	186.94	196.90	196.01	6.07	3.1%
7.97_483.1822m/z	483.1822	7.97	198.4	30.97	27.93	32.02	31.66	31.00	30.91	30.75	1.32	4.3%
7.97_784.3413m/z	784.3413	7.97	260.3	54.30	51.94	53.65	59.40	49.17	54.35	53.80	3.08	5.7%
7.96_438.1955m/z	438.1955	7.96	202.9	26.53	25.50	26.88	26.40	27.14	26.49	26.49	0.51	1.9%
7.96_456.2094m/z	456.2094	7.96	202.5	300.34	294.57	302.25	300.52	303.04	300.10	300.14	2.71	0.9%
7.88_499.2512m/z	499.2512	7.88	210.6	648.23	636.31	652.81	648.58	653.06	647.71	647.78	5.56	0.9%
7.87_676.2910m/z	676.2910	7.87	237.1	76.72	71.72	80.83	74.18	79.33	76.54	76.56	3.03	4.0%
7.87_613.2945m/z	613.2945	7.87	228.7	1311.60	1292.09	1307.79	1352.53	1277.13	1311.63	1308.80	23.14	1.8%

7.87_454.2299m/z	454.2299	7.87	202.6	1330.82	1312.77	1344.41	1328.87	1331.17	1330.17	1329.70	9.20	0.7%
7.87_428.2145m/z	428.2145	7.87	197.8	2526.98	2474.63	2544.99	2557.82	2509.02	2525.96	2523.23	26.63	1.1%
7.86_219.0762m/z	219.0762	7.86	148.3	36.30	36.22	35.39	36.77	36.84	36.27	36.30	0.47	1.3%
7.86_632.2687m/z	632.2687	7.86	235.9	796.93	786.87	798.54	793.48	809.45	796.12	796.90	6.75	0.8%
7.86_779.8748m/z	779.8748	7.86	208.3	396.94	380.14	391.56	434.84	361.93	397.19	393.76	22.03	5.6%
7.86_714.3135m/z	714.3135	7.86	194.9	107.09	105.37	104.91	115.42	98.45	107.18	106.40	4.98	4.7%
7.92_275.1105n	256.0931	7.92	159.9	263.33	255.17	274.59	245.61	277.05	262.76	263.09	10.76	4.1%
7.92_401.2036m/z	401.2036	7.92	194.9	529.80	520.63	548.35	507.88	539.24	529.34	529.21	12.88	2.4%
7.91_681.3195m/z	681.3195	7.91	246.4	110.33	107.98	113.94	103.04	117.23	109.89	110.40	4.46	4.0%
7.91_464.2241m/z	464.2241	7.91	213.2	39.53	36.33	43.75	35.04	41.43	39.44	39.25	2.92	7.5%
7.91_713.8125m/z	713.8125	7.91	194.9	671.36	664.56	667.94	680.88	669.11	671.10	670.83	5.03	0.8%
7.90_681.3223m/z	681.3223	7.90	246.4	27.67	24.58	30.51	27.42	26.43	27.66	27.38	1.77	6.4%
7.90_358.1943m/z	358.1943	7.90	187.3	37.94	36.82	41.00	29.97	42.73	37.67	37.69	4.02	10.7%
7.90_337.0430m/z	337.0430	7.90	165.5	25.98	23.53	28.00	24.12	27.38	25.91	25.82	1.60	6.2%
7.90_611.2798m/z	611.2798	7.90	230.6	528.78	528.87	531.21	503.83	557.00	527.53	529.54	15.40	2.9%
7.90_241.0827m/z	241.0827	7.90	152.2	490.95	488.78	497.02	475.70	503.80	490.46	491.12	8.56	1.7%
7.74_258.1450m/z	258.1450	7.74	163.2	466.54	462.01	469.28	470.78	460.80	466.47	465.98	3.58	0.8%
7.56_529.2611m/z	529.2611	7.56	217.3	584.32	591.49	572.09	590.27	584.49	584.13	584.46	6.27	1.1%
7.60_649.7851m/z	649.7851	7.60	185.3	2145.12	2116.99	2142.79	2196.89	2101.53	2144.96	2141.38	29.70	1.4%
7.59_582.2866m/z	582.2866	7.59	223.7	4513.27	4512.89	4465.39	4630.19	4406.46	4513.90	4507.02	67.25	1.5%
7.57_586.2821m/z	586.2821	7.57	223.7	194.99	192.14	192.78	208.39	179.18	195.16	193.77	8.50	4.4%
7.52_369.1411m/z	369.1411	7.52	183.5	823.05	821.97	818.55	830.72	818.88	822.84	822.67	4.02	0.5%
7.49_958.4282m/z	958.4282	7.49	287.5	331.87	334.61	312.11	347.55	331.09	331.74	331.50	10.36	3.1%
7.52_650.2865m/z	650.2865	7.52	185.3	1166.42	1167.71	1142.63	1174.65	1189.10	1164.62	1167.52	13.82	1.2%
7.55_315.1667m/z	315.1667	7.55	173.0	529.51	525.41	522.49	541.81	526.18	529.26	529.11	6.16	1.2%
7.55_555.2777m/z	555.2777	7.55	224.2	1701.01	1707.98	1644.72	1771.16	1667.53	1701.12	1698.92	39.23	2.3%
7.55_241.0829m/z	241.0829	7.55	152.2	2147.83	2119.02	2185.50	2113.79	2165.76	2146.44	2146.39	24.90	1.2%
7.54_426.1626m/z	426.1626	7.54	192.6	1090.46	1099.58	1064.66	1089.90	1119.45	1088.54	1092.10	16.21	1.5%
7.54_653.3262m/z	653.3262	7.54	237.4	2712.42	2697.19	2684.97	2806.43	2627.72	2712.97	2706.95	52.94	2.0%

7.53_197.0926m/z	197.0926	7.53	141.3	14.29	12.60	15.18	14.53	14.12	0.00	11.79	5.33	45.2%
7.52_683.3351m/z	683.3351	7.52	244.5	926.73	914.44	921.27	954.68	905.72	926.68	924.92	15.19	1.6%
7.60_728.1511m/z	728.1511	7.60	247.7	161.46	165.33	164.66	149.58	161.24	0.00	133.71	60.02	44.9%
7.71_288.1549m/z	288.1549	7.71	170.5	196.58	190.64	201.59	191.28	202.16	196.27	196.42	4.46	2.3%
7.71_488.2135m/z	488.2135	7.71	210.9	269.47	267.34	272.95	263.21	275.10	269.17	269.54	3.82	1.4%
7.71_606.7608m/z	606.7608	7.71	182.4	964.14	962.37	945.79	979.68	968.55	963.58	964.02	10.01	1.0%
7.70_614.7665m/z	614.7665	7.70	180.6	230.91	224.08	229.32	241.60	223.48	230.89	230.05	5.98	2.6%
7.70_477.1722m/z	477.1722	7.70	202.1	430.72	432.39	427.03	433.41	429.77	430.62	430.66	2.02	0.5%
7.69_408.0887m/z	408.0887	7.69	203.7	227.51	216.02	231.98	225.15	244.61	226.76	228.67	8.59	3.8%
7.72_452.1782m/z	452.1782	7.72	195.5	700.50	697.41	694.53	706.57	702.55	700.16	700.29	3.79	0.5%
7.71_667.2680m/z	667.2680	7.71	237.2	348.10	348.23	341.05	346.61	359.26	347.52	348.46	5.42	1.6%
7.60_319.0761m/z	319.0761	7.60	183.2	7.75	6.69	8.81	0.00	8.12	0.00	5.23	3.75	71.7%
7.67_614.2658m/z	614.2658	7.67	180.6	641.41	632.77	640.52	653.22	634.24	641.24	640.57	6.61	1.0%
7.66_327.1666m/z	327.1666	7.66	172.6	755.81	745.92	755.10	769.82	746.61	755.63	754.82	7.89	1.0%
7.66_590.2577m/z	590.2577	7.66	225.4	1272.49	1276.45	1248.79	1301.70	1258.09	1272.39	1271.65	16.50	1.3%
8.00_541.2553m/z	541.2553	8.00	222.6	20.73	20.41	21.56	18.99	22.03	20.67	20.73	0.96	4.6%
8.37_520.2046m/z	520.2046	8.37	217.5	1692.18	1761.49	1679.29	1659.63	1688.13	1694.67	1695.90	31.54	1.9%
8.36_373.1753m/z	373.1753	8.36	186.9	0.00	12.07	12.76	10.99	13.04	12.22	10.18	4.60	45.2%
8.36_359.1570m/z	359.1570	8.36	178.6	40.03	40.84	41.53	38.68	40.86	40.41	40.39	0.89	2.2%
8.36_548.0988m/z	548.0988	8.36	215.2	25.50	25.14	27.82	24.25	25.83	25.92	25.75	1.08	4.2%
8.36_543.2387m/z	543.2387	8.36	218.9	157.51	166.83	154.95	156.07	157.94	158.47	158.63	3.85	2.4%
8.36_796.3456m/z	796.3456	8.36	264.0	534.44	576.41	540.60	535.13	548.44	548.03	547.17	14.19	2.6%
8.36_486.2204m/z	486.2204	8.36	210.9	7584.07	7854.57	7458.17	7580.39	7523.02	7593.06	7598.88	123.51	1.6%
8.35_627.2730m/z	627.2730	8.35	230.4	134.05	142.07	131.24	132.89	134.93	134.80	135.00	3.40	2.5%
8.35_399.1873m/z	399.1873	8.35	193.2	74.11	79.09	72.98	74.35	70.95	74.65	74.36	2.45	3.3%
8.35_299.1349m/z	299.1349	8.35	165.0	105.25	107.72	106.90	103.26	103.19	105.42	105.29	1.68	1.6%
8.34_415.1826m/z	415.1826	8.34	192.8	92.37	96.93	94.06	85.71	96.26	92.91	93.04	3.67	3.9%
8.34_300.1536m/z	300.1536	8.34	170.1	177.30	180.28	180.84	171.86	176.44	177.42	177.36	2.94	1.7%
8.34_430.1941m/z	430.1941	8.34	197.8	285.71	304.38	283.29	278.83	290.94	288.26	288.57	8.03	2.8%

8.37_566.2570m/z	566.2570	8.37	224.0	767.18	794.85	758.90	760.31	761.99	768.06	768.55	12.23	1.6%
8.42_302.1352m/z	302.1352	8.42	168.3	531.85	571.50	511.16	531.20	536.02	535.33	536.18	17.88	3.3%
8.42_577.2276m/z	577.2276	8.42	222.0	1178.40	1227.88	1151.83	1187.57	1160.04	1180.48	1181.03	24.29	2.1%
8.41_392.5753n	784.1496	8.41	239.4	1180.82	1249.11	1143.21	1193.00	1169.35	1185.77	1186.88	32.05	2.7%
8.41_432.5752m/z	432.5752	8.41	150.1	447.00	463.04	437.61	448.17	443.28	447.42	447.75	7.72	1.7%
8.40_476.7443m/z	476.7443	8.40	162.4	25.55	24.55	26.84	25.26	24.94	25.56	25.45	0.71	2.8%
8.39_788.3242m/z	788.3242	8.39	266.0	15.05	20.25	19.80	18.15	20.37	19.52	18.86	1.85	9.8%
8.39_304.0319m/z	304.0319	8.39	161.5	55.49	54.54	57.15	54.98	54.83	55.49	55.41	0.85	1.5%
8.39_386.1649m/z	386.1649	8.39	184.8	78.66	81.42	75.43	81.48	76.43	78.74	78.69	2.27	2.9%
8.39_699.2949m/z	699.2949	8.39	246.2	660.20	690.51	650.60	658.35	653.11	662.04	662.47	13.15	2.0%
8.37_330.1657m/z	330.1657	8.37	181.1	353.78	389.53	351.98	357.71	364.32	364.40	363.62	12.52	3.4%
8.37_302.1352m/z	302.1352	8.37	168.3	133.81	141.80	130.78	130.57	134.04	134.10	134.18	3.71	2.8%
8.42_242.1105m/z	242.1105	8.42	157.2	8.34	8.76	8.03	8.28	8.39	8.35	8.36	0.21	2.6%
8.31_600.2712m/z	600.2712	8.31	180.8	214.18	218.24	214.00	217.28	205.67	214.42	213.96	4.05	1.9%
8.30_463.1829m/z	463.1829	8.30	202.4	12.76	13.50	12.62	12.13	12.95	12.78	12.79	0.41	3.2%
8.30_510.2521m/z	510.2521	8.30	221.4	50.58	52.23	52.19	46.42	51.16	50.64	50.54	1.95	3.9%
8.30_495.1832m/z	495.1832	8.30	207.1	93.52	99.58	90.13	93.91	92.16	93.83	93.85	2.87	3.1%
8.30_637.3091m/z	637.3091	8.30	183.7	369.78	374.43	369.48	368.04	367.81	369.84	369.90	2.18	0.6%
8.29_822.1355m/z	822.1355	8.29	259.8	0.00	20.41	19.38	21.73	20.17	20.54	17.04	7.65	44.9%
8.29_392.1663m/z	392.1663	8.29	188.1	43.08	44.17	43.55	43.09	41.44	43.17	43.08	0.83	1.9%
8.31_746.7861m/z	746.7861	8.31	196.3	45.21	45.37	45.50	46.49	42.48	45.29	45.06	1.23	2.7%
8.33_844.8618m/z	844.8618	8.33	214.8	44.39	45.80	45.75	39.96	46.40	44.42	44.45	2.14	4.8%
8.33_517.2260m/z	517.2260	8.33	210.3	1175.29	1226.55	1162.59	1165.52	1167.09	1178.38	1179.24	21.86	1.9%
8.32_713.3059m/z	713.3059	8.32	240.3	0.00	5.28	5.62	5.32	5.19	5.37	4.46	2.00	44.8%
8.32_611.1308m/z	611.1308	8.32	230.6	14.09	14.47	14.64	12.07	15.01	14.08	14.06	0.94	6.7%
8.32_251.0771m/z	251.0771	8.32	146.9	29.91	32.42	28.58	30.48	29.64	30.21	30.21	1.16	3.8%
8.32_444.2291m/z	444.2291	8.32	166.4	993.48	1057.70	969.84	938.42	1020.67	994.85	995.83	37.47	3.8%
8.32_356.1836m/z	356.1836	8.32	178.6	1.92	2.17	1.68	2.20	1.53	1.96	1.91	0.24	12.7%
8.32_564.2342m/z	564.2342	8.32	169.4	17.90	19.50	17.08	16.70	18.77	17.96	17.99	0.95	5.3%

8.32_509.2342m/z	509.2342	8.32	215.9	107.20	105.54	112.09	103.06	106.80	107.18	106.98	2.70	2.5%
8.32_749.3574m/z	749.3574	8.32	260.7	818.34	837.00	803.17	829.89	804.57	818.78	818.62	12.25	1.5%
8.31_325.1498m/z	325.1498	8.31	174.4	5.78	5.09	6.51	5.28	5.79	5.77	5.70	0.45	7.9%
8.29_559.2349m/z	559.2349	8.29	222.3	454.42	464.39	454.18	447.44	456.20	454.69	455.22	4.96	1.1%
8.53_586.2463m/z	586.2463	8.53	223.7	48.50	53.00	49.25	47.71	50.81	49.98	49.88	1.72	3.4%
8.53_865.3691m/z	865.3691	8.53	272.8	1378.78	1475.84	1345.06	1409.20	1365.00	1395.76	1394.94	41.62	3.0%
8.52_685.2708m/z	685.2708	8.52	237.0	23.03	28.85	25.34	27.79	25.85	26.92	26.30	1.87	7.1%
8.52_713.3136m/z	713.3136	8.52	244.1	55.13	65.69	45.94	60.59	56.53	57.48	56.89	5.98	10.5%
8.52_489.1936m/z	489.1936	8.52	200.1	251.62	255.53	252.59	255.54	242.93	252.14	251.73	4.23	1.7%
8.51_185.0921m/z	185.0921	8.51	143.6	36.10	35.09	37.59	34.66	36.73	36.07	36.04	0.97	2.7%
8.51_399.1491m/z	399.1491	8.51	184.5	89.50	93.75	90.84	88.59	91.55	90.83	90.84	1.62	1.8%
8.51_753.3413m/z	753.3413	8.51	253.0	836.69	877.97	817.41	843.94	826.00	839.76	840.30	19.04	2.3%
8.50_371.1548m/z	371.1548	8.50	183.4	9.41	9.80	9.49	9.15	9.54	9.46	9.47	0.19	2.0%
8.50_359.1525m/z	359.1525	8.50	180.3	49.40	51.62	47.48	50.39	48.78	49.48	49.53	1.28	2.6%
8.50_638.2769m/z	638.2769	8.50	187.2	1079.86	1116.47	1047.84	1108.13	1056.98	1081.52	1081.80	24.73	2.3%
8.57_752.8215m/z	752.8215	8.57	198.0	24.10	26.85	20.86	26.23	22.64	24.31	24.17	2.03	8.4%
8.56_346.2253m/z	346.2253	8.56	172.1	78.27	90.21	83.54	80.29	88.21	84.96	84.25	4.16	4.9%
8.56_359.1537m/z	359.1537	8.56	178.6	22.97	23.92	23.73	20.67	23.31	23.01	22.93	1.07	4.7%
8.56_752.3189m/z	752.3189	8.56	194.5	111.65	120.70	110.53	107.83	117.62	113.42	113.62	4.34	3.8%
8.47_403.5544m/z	403.5544	8.47	145.8	1037.37	1061.02	1023.70	1034.88	1035.23	1037.61	1038.30	11.20	1.1%
8.46_357.1396m/z	357.1396	8.46	176.9	109.85	117.76	106.77	111.40	107.09	110.69	110.60	3.64	3.3%
8.46_541.2254m/z	541.2254	8.46	217.1	338.85	360.61	330.77	340.13	336.83	341.12	341.39	9.23	2.7%
8.46_940.3639m/z	940.3639	8.46	281.8	436.83	548.69	508.98	498.54	513.28	515.89	503.70	33.64	6.7%
8.46_463.1825m/z	463.1825	8.46	200.6	426.71	443.89	416.67	429.67	422.24	427.39	427.76	8.35	2.0%
8.46_245.0431m/z	245.0431	8.46	147.1	657.56	673.61	650.65	650.41	661.21	657.72	658.53	7.79	1.2%
8.45_404.0554m/z	404.0554	8.45	145.8	239.81	250.86	232.19	243.11	235.09	240.18	240.21	5.95	2.5%
8.45_458.1120n	413.1149	8.45	189.4	234.83	256.87	243.65	236.12	248.97	245.15	244.26	7.50	3.1%
8.44_616.2567m/z	616.2567	8.44	226.9	298.32	318.31	287.84	296.38	302.32	299.85	300.50	9.16	3.0%
8.43_360.1678m/z	360.1678	8.43	182.0	0.00	6.21	6.20	6.84	5.50	6.30	5.18	2.35	45.3%

8.47_468.1736m/z	468.1736	8.47	202.3	350.24	368.77	352.75	349.25	351.05	354.53	354.43	6.64	1.9%
8.47_253.0814m/z	253.0814	8.47	156.7	56.83	55.22	59.35	55.64	56.34	56.82	56.70	1.32	2.3%
8.49_469.6772m/z	469.6772	8.49	162.6	381.07	395.73	372.46	379.70	383.93	381.65	382.42	6.94	1.8%
8.49_429.1982m/z	429.1982	8.49	196.0	922.98	955.92	907.93	922.55	913.97	923.98	924.56	15.16	1.6%
8.48_346.1571m/z	346.1571	8.48	178.9	16.62	17.94	15.75	16.42	16.46	16.66	16.64	0.65	3.9%
8.48_191.0550m/z	191.0550	8.48	135.1	72.57	73.91	73.56	70.32	73.26	72.63	72.71	1.17	1.6%
8.48_638.7777m/z	638.7777	8.48	188.9	582.45	601.92	567.35	594.81	573.31	583.56	583.90	11.77	2.0%
8.48_270.1089m/z	270.1089	8.48	157.7	369.64	383.38	369.53	360.39	372.15	370.41	370.92	6.73	1.8%
8.47_373.1699m/z	373.1699	8.47	183.4	47.88	50.66	47.36	45.21	47.92	47.93	47.83	1.59	3.3%
8.47_608.2666m/z	608.2666	8.47	228.8	30.85	33.00	32.53	31.23	31.60	32.07	31.88	0.74	2.3%
8.47_483.2180m/z	483.2180	8.47	207.4	277.16	291.37	267.94	279.36	274.91	277.76	278.08	6.98	2.5%
8.47_269.0620m/z	269.0620	8.47	152.7	120.18	126.53	112.20	121.39	122.55	120.15	120.50	4.29	3.6%
8.11_722.8415m/z	722.8415	8.11	198.3	19.15	20.92	16.37	20.17	19.04	19.18	19.14	1.41	7.4%
8.11_400.1470m/z	400.1470	8.11	181.0	136.83	138.69	132.42	142.39	132.92	136.88	136.69	3.39	2.5%
8.11_573.7549m/z	573.7549	8.11	174.3	564.43	566.30	559.82	567.66	563.61	564.30	564.35	2.44	0.4%
8.11_474.1730m/z	474.1730	8.11	202.2	395.66	397.98	394.66	393.79	396.89	395.56	395.76	1.38	0.3%
8.10_709.2766m/z	709.2766	8.10	240.4	25.86	28.10	21.78	29.06	23.51	25.94	25.71	2.49	9.7%
8.10_567.2758m/z	567.2758	8.10	227.7	691.64	693.53	686.33	700.84	684.00	691.64	691.33	5.39	0.8%
8.09_679.2995m/z	679.2995	8.09	240.8	31.36	30.78	32.86	28.59	33.25	31.27	31.35	1.52	4.8%
8.09_510.2526m/z	510.2526	8.09	217.7	169.87	173.56	168.24	162.41	176.60	169.71	170.07	4.42	2.6%
8.09_628.2907m/z	628.2907	8.09	235.9	140.82	141.00	141.78	138.09	142.53	140.74	140.83	1.37	1.0%
8.09_841.8739m/z	841.8739	8.09	213.0	92.93	94.09	92.78	89.64	96.19	92.85	93.08	1.95	2.1%
8.08_709.3130m/z	709.3130	8.08	195.0	30.74	29.15	32.36	30.09	30.71	30.71	30.63	0.96	3.1%
8.08_509.2011m/z	509.2011	8.08	214.1	54.72	54.98	54.64	55.72	53.20	54.75	54.67	0.75	1.4%
8.08_444.2097m/z	444.2097	8.08	199.2	2040.23	2050.74	2004.25	2094.99	2000.18	2040.53	2038.49	31.59	1.5%
8.16_638.2998n	637.2926	8.16	235.8	598.27	608.97	590.13	598.39	598.65	598.31	598.79	5.47	0.9%
8.16_272.0880m/z	272.0880	8.16	159.3	240.29	244.67	239.52	237.16	240.57	240.31	240.42	2.22	0.9%
8.15_316.1489m/z	316.1489	8.15	169.6	39.96	42.86	36.90	40.58	40.07	40.03	40.07	1.74	4.3%
8.15_709.2805m/z	709.2805	8.15	247.9	30.04	30.47	29.05	30.72	30.02	30.03	30.05	0.52	1.7%

8.15_518.2249m/z	518.2249	8.15	219.4	219.01	222.94	217.43	214.33	222.95	218.92	219.27	3.03	1.4%
8.15_387.1862m/z	387.1862	8.15	188.2	239.27	243.13	240.75	235.50	238.55	239.36	239.43	2.30	1.0%
8.14_582.1903m/z	582.1903	8.14	225.6	25.83	23.82	27.84	23.38	27.56	25.73	25.69	1.68	6.5%
8.13_413.1996m/z	413.1996	8.13	192.9	38.90	41.37	34.06	42.68	36.68	38.97	38.78	2.85	7.3%
8.13_688.3065m/z	688.3065	8.13	195.2	340.97	327.77	346.85	353.29	326.99	340.99	339.48	9.51	2.8%
8.13_391.1615m/z	391.1615	8.13	189.9	434.45	430.50	444.00	421.64	440.81	434.15	434.26	7.20	1.7%
8.12_345.1887n	344.1813	8.12	184.2	380.33	383.57	386.35	366.51	385.05	380.21	380.34	6.59	1.7%
8.12_920.4097m/z	920.4097	8.12	280.0	798.31	809.50	777.22	821.36	782.71	798.53	797.94	14.96	1.9%
8.16_807.3269m/z	807.3269	8.16	265.8	27.41	26.71	29.16	24.67	28.79	27.37	27.35	1.47	5.4%
8.03_399.1880m/z	399.1880	8.03	191.4	134.08	134.25	134.36	132.52	135.68	134.00	134.15	0.92	0.7%
8.03_640.2932m/z	640.2932	8.03	237.6	447.55	444.75	450.40	447.88	445.51	447.44	447.25	1.81	0.4%
8.03_642.3094m/z	642.3094	8.03	233.9	664.73	663.41	656.73	677.36	658.84	664.62	664.28	6.57	1.0%
8.01_575.2442m/z	575.2442	8.01	229.4	53.11	50.93	54.99	53.25	52.25	53.09	52.94	1.22	2.3%
8.01_217.1189m/z	217.1189	8.01	151.7	494.52	487.32	502.63	483.89	503.59	494.07	494.34	7.22	1.5%
8.01_287.1242m/z	287.1242	8.01	167.1	36.18	36.37	36.75	32.84	39.24	35.99	36.23	1.87	5.2%
8.01_591.2395m/z	591.2395	8.01	227.3	32.63	30.68	35.91	26.10	36.55	32.41	32.38	3.47	10.7%
8.03_141.0663m/z	141.0663	8.03	130.7	16.35	16.28	16.32	16.14	16.66	16.34	16.35	0.15	0.9%
8.04_841.3723m/z	841.3723	8.04	209.4	544.21	547.52	531.55	566.02	526.19	544.42	543.32	12.69	2.3%
8.06_541.2549m/z	541.2549	8.06	217.1	12.51	12.56	12.85	10.52	13.97	12.44	12.48	1.02	8.2%
8.06_454.2318m/z	454.2318	8.06	199.0	15.03	15.93	14.38	14.04	16.03	15.01	15.07	0.73	4.8%
8.06_584.2668m/z	584.2668	8.06	225.5	35.14	36.43	33.40	33.94	37.22	35.07	35.20	1.32	3.7%
8.06_650.2810m/z	650.2810	8.06	188.7	502.02	504.09	493.95	510.26	498.83	501.95	501.85	4.95	1.0%
8.06_227.1031m/z	227.1031	8.06	152.9	429.09	427.62	436.41	418.50	432.97	428.89	428.91	5.52	1.3%
8.06_486.2320m/z	486.2320	8.06	209.1	9.48	9.64	9.75	9.25	8.92	9.50	9.42	0.27	2.9%
8.06_673.2835m/z	673.2835	8.06	188.4	105.41	104.42	107.13	102.98	107.04	105.34	105.39	1.44	1.4%
8.06_455.0959m/z	455.0959	8.06	199.0	165.75	164.16	171.18	158.05	169.34	165.62	165.68	4.17	2.5%
8.06_199.0374m/z	199.0374	8.06	134.7	157.51	156.29	155.47	162.91	153.72	157.52	157.24	2.85	1.8%
8.05_893.3785m/z	893.3785	8.05	284.3	68.00	70.94	64.88	68.15	68.73	68.01	68.12	1.77	2.6%
8.05_261.0872m/z	261.0872	8.05	159.7	108.35	107.23	112.40	103.77	109.26	108.30	108.22	2.56	2.4%

8.04_265.0930m/z	265.0930	8.04	161.2	187.66	181.90	194.67	179.35	193.79	187.38	187.46	5.61	3.0%
8.04_185.0566m/z	185.0566	8.04	137.1	752.00	742.20	767.23	736.05	759.87	751.52	751.48	10.35	1.4%
8.26_615.2049m/z	615.2049	8.26	226.9	26.22	27.45	24.97	27.07	25.58	26.27	26.26	0.83	3.2%
8.26_616.7600m/z	616.7600	8.26	189.2	71.35	73.04	70.54	71.42	70.91	71.39	71.44	0.78	1.1%
8.26_472.2033m/z	472.2033	8.26	202.2	157.47	161.20	156.18	157.97	155.18	157.59	157.60	1.87	1.2%
8.26_583.2724m/z	583.2724	8.26	229.2	857.33	880.85	839.90	869.91	842.31	858.06	858.06	14.36	1.7%
8.25_681.2896m/z	681.2896	8.25	193.6	0.00	4.42	5.10	3.90	4.54	4.57	3.76	1.72	45.7%
8.25_552.2190m/z	552.2190	8.25	215.1	15.21	15.80	14.98	15.81	14.08	15.30	15.20	0.58	3.8%
8.25_399.0963m/z	399.0963	8.25	174.1	48.92	24.13	88.55	65.54	50.24	48.79	54.36	19.52	35.9%
8.24_527.2458m/z	527.2458	8.24	215.5	160.97	172.31	154.41	163.96	157.99	161.93	161.93	5.55	3.4%
8.24_410.5617m/z	410.5617	8.24	145.7	549.89	561.34	539.89	557.05	542.72	550.13	550.17	7.45	1.4%
8.24_600.2632m/z	600.2632	8.24	225.3	982.71	1002.51	971.59	988.03	972.08	983.16	983.35	10.45	1.1%
8.27_610.2477m/z	610.2477	8.27	226.9	508.75	516.42	508.12	509.11	503.05	509.06	509.08	3.90	0.8%
8.27_383.1219m/z	383.1219	8.27	174.5	420.10	414.90	424.50	426.05	409.13	420.13	419.14	5.72	1.4%
8.27_370.1705m/z	370.1705	8.27	178.3	24.94	23.57	27.23	22.77	25.23	24.92	24.78	1.40	5.6%
8.28_329.1096m/z	329.1096	8.28	167.5	77.30	81.31	76.73	74.47	78.28	77.50	77.60	2.04	2.6%
8.28_216.0869m/z	216.0869	8.28	150.1	80.32	87.08	82.16	83.29	77.81	83.01	82.28	2.84	3.5%
8.28_599.7705m/z	599.7705	8.28	180.8	505.95	508.11	510.17	510.05	491.24	506.23	505.29	6.50	1.3%
8.27_608.2661m/z	608.2661	8.27	230.7	11.95	13.87	11.20	11.20	13.20	12.37	12.30	0.99	8.0%
8.27_484.2353m/z	484.2353	8.27	212.8	20.18	21.19	19.41	20.98	19.38	20.26	20.24	0.69	3.4%
8.19_259.1290m/z	259.1290	8.19	159.8	211.81	219.40	210.48	209.73	208.97	212.08	212.08	3.45	1.6%
8.19_300.1554m/z	300.1554	8.19	175.2	1272.51	1277.01	1266.81	1280.53	1263.28	1272.41	1272.09	5.79	0.5%
8.19_357.1777m/z	357.1777	8.19	182.1	2696.84	2714.45	2713.60	2664.18	2695.25	2696.58	2696.82	16.64	0.6%
8.18_373.1721m/z	373.1721	8.18	186.9	34.16	36.55	32.19	33.69	35.30	34.22	34.35	1.35	3.9%
8.19_303.1425n	284.1247	8.19	163.9	1773.27	1794.99	1772.39	1767.19	1759.76	1773.59	1773.53	10.74	0.6%
8.17_551.2408m/z	551.2408	8.17	226.1	25.89	26.67	24.94	26.08	25.99	25.89	25.91	0.51	2.0%
8.17_438.1979m/z	438.1979	8.17	201.2	43.06	44.53	41.31	44.16	42.41	43.09	43.09	1.07	2.5%
8.16_486.2147m/z	486.2147	8.16	205.5	12.15	12.27	12.37	12.87	10.59	12.21	12.08	0.71	5.8%
8.16_320.1264m/z	320.1264	8.16	179.7	52.09	54.06	49.02	54.30	50.84	52.12	52.07	1.82	3.5%

8.16_267.0967m/z	267.0967	8.16	161.1	72.84	73.72	70.98	75.30	70.99	72.87	72.79	1.51	2.1%
8.16_624.2990m/z	624.2990	8.16	234.1	1234.07	1251.68	1212.54	1255.18	1215.99	1234.37	1233.97	16.05	1.3%
8.23_371.1928m/z	371.1928	8.23	186.9	5.47	5.84	5.24	4.87	6.01	5.45	5.48	0.37	6.8%
8.23_313.1508m/z	313.1508	8.23	171.4	209.09	213.57	205.84	213.76	202.05	209.25	208.93	4.12	2.0%
8.23_542.7549m/z	542.7549	8.23	176.5	330.95	320.00	334.89	339.83	323.84	330.87	330.07	6.58	2.0%
8.22_352.0637m/z	352.0637	8.22	173.6	166.77	167.89	169.66	162.75	166.68	166.78	166.75	2.07	1.2%
8.22_281.1129m/z	281.1129	8.22	165.7	11.69	11.72	11.87	11.53	11.67	11.69	11.69	0.10	0.9%
8.22_265.0944m/z	265.0944	8.22	156.2	22.20	22.70	23.10	19.88	23.04	22.20	22.19	1.09	4.9%
8.22_754.3392m/z	754.3392	8.22	260.7	108.93	115.26	105.91	107.09	109.71	109.17	109.34	2.95	2.7%
8.22_904.4152m/z	904.4152	8.22	282.2	812.74	834.56	800.89	813.61	807.02	813.20	813.67	10.37	1.3%
8.21_513.2270m/z	513.2270	8.21	215.8	46.19	47.61	46.43	45.88	45.02	46.28	46.24	0.77	1.7%
8.21_784.3445m/z	784.3445	8.21	254.5	187.42	192.36	183.85	186.80	188.82	187.47	187.79	2.54	1.4%
8.20_549.0825m/z	549.0825	8.20	217.0	15.53	15.35	16.51	15.10	15.06	15.62	15.53	0.48	3.1%
10.26_96.9601m/z	96.9601	10.26	111.9	1276.21	1292.39	1272.72	1265.65	1275.70	1276.16	1276.47	8.01	0.6%
11.19_416.9487m/z	416.9487	11.19	170.3	142.31	144.05	143.19	140.31	140.57	142.33	142.13	1.33	0.9%
11.25_326.1683m/z	326.1683	11.25	183.0	57.31	56.92	48.87	64.29	54.27	57.36	56.50	4.57	8.1%
11.25_544.9681n	589.9577	11.25	198.3	410.57	411.29	432.96	367.88	414.77	410.47	407.99	19.59	4.8%
11.22_422.9619m/z	422.9619	11.22	166.8	1901.28	2001.67	1811.18	1854.89	1894.53	1902.24	1894.30	57.88	3.1%
11.27_394.9670m/z	394.9670	11.27	159.1	2050.57	2155.27	1948.59	2021.69	2027.08	2051.73	2042.49	61.04	3.0%
11.81_370.2092m/z	370.2092	11.81	190.5	797.24	795.88	796.17	808.49	783.29	797.27	796.39	7.30	0.9%
11.79_524.2836m/z	524.2836	11.79	221.1	223.50	224.19	221.65	225.01	222.93	223.46	223.46	1.04	0.5%
11.76_438.1954m/z	438.1954	11.76	202.9	62.86	62.30	61.48	64.27	63.10	62.83	62.81	0.84	1.3%
11.76_308.1612m/z	308.1612	11.76	176.7	73.69	74.26	72.89	74.80	72.55	73.70	73.65	0.76	1.0%
11.73_315.2033m/z	315.2033	11.73	185.1	292.42	293.63	292.30	292.57	290.56	292.40	292.31	0.90	0.3%
11.63_244.1664m/z	244.1664	11.63	162.1	363.94	356.40	373.00	352.41	373.07	363.52	363.72	7.69	2.1%
11.81_343.2087m/z	343.2087	11.81	186.0	61.88	62.04	61.93	61.51	61.99	61.86	61.87	0.17	0.3%
11.88_260.1351m/z	260.1351	11.88	159.7	20.08	20.62	19.75	20.60	19.29	20.10	20.07	0.46	2.3%
11.96_331.1982m/z	331.1982	11.96	184.6	294.38	307.56	283.32	311.42	271.90	294.95	293.92	13.48	4.6%
11.93_398.2153m/z	398.2153	11.93	191.5	227.69	233.73	223.70	233.24	218.64	227.89	227.48	5.24	2.3%

11.90_552.2901m/z	552.2901	11.90	222.4	214.21	212.74	215.93	217.67	207.88	214.26	213.78	3.06	1.4%
11.83_232.1297m/z	232.1297	11.83	157.6	43.43	44.88	41.55	44.45	42.94	43.45	43.45	1.07	2.5%
11.37_426.9635m/z	426.9635	11.37	165.1	402.43	424.58	381.16	393.96	399.73	402.67	400.75	12.94	3.2%
11.32_244.1663m/z	244.1663	11.32	165.5	271.33	276.17	264.07	277.83	267.07	271.41	271.31	4.77	1.8%
11.57_758.3439m/z	758.3439	11.57	201.4	460.67	457.53	466.94	455.47	460.73	460.50	460.31	3.54	0.8%
11.51_315.0382m/z	315.0382	11.51	159.5	4.46	4.61	4.46	4.42	4.29	4.47	4.45	0.09	2.1%
11.45_888.4638m/z	888.4638	11.45	310.4	664.66	691.36	706.71	525.58	693.80	666.23	658.06	61.12	9.3%
11.44_526.2925m/z	526.2925	11.44	222.9	2.35	2.39	2.37	2.05	2.60	2.34	2.35	0.16	6.9%
11.40_398.9686m/z	398.9686	11.40	162.3	424.06	448.51	395.50	425.58	417.00	424.37	422.50	15.55	3.7%
11.48_232.1298m/z	232.1298	11.48	155.9	85.52	86.92	84.26	86.72	84.02	85.54	85.50	1.10	1.3%
9.47_203.1035m/z	203.1035	9.47	145.8	707.08	703.40	731.65	759.90	547.75	708.51	693.05	67.84	9.8%
10.08_424.9765m/z	424.9765	10.08	177.0	302.24	304.92	277.17	314.11	307.47	302.00	301.32	11.54	3.8%
10.08_315.1299m/z	315.1299	10.08	179.9	41.27	36.24	42.81	44.35	43.54	41.17	41.56	2.64	6.4%
10.14_317.1458m/z	317.1458	10.14	167.8	34.18	34.93	30.67	34.39	36.29	34.12	34.10	1.70	5.0%
10.09_305.5685m/z	305.5685	10.09	137.0	104.05	103.99	101.57	105.33	105.14	103.99	104.01	1.22	1.2%
10.09_305.0678m/z	305.0678	10.09	135.4	465.37	466.61	454.08	469.58	470.87	465.09	465.27	5.43	1.2%
9.72_309.0097m/z	309.0097	9.72	156.3	174.38	184.77	168.49	161.11	184.53	174.29	174.60	8.38	4.8%
9.70_316.1131m/z	316.1131	9.70	167.9	23.16	20.11	24.26	22.82	24.25	23.09	22.95	1.39	6.1%
9.78_639.3215m/z	639.3215	9.78	239.5	1051.47	1039.64	970.83	1116.04	1059.01	1050.59	1047.93	42.42	4.0%
9.66_362.0505m/z	362.0505	9.66	168.2	579.52	574.15	559.82	577.06	605.02	578.44	579.00	13.37	2.3%
9.64_335.0474m/z	335.0474	9.64	157.2	998.34	996.81	981.44	948.50	1064.26	995.92	997.55	34.44	3.5%
9.92_382.5488m/z	382.5488	9.92	141.4	593.97	588.78	581.88	601.01	603.49	593.46	593.77	7.23	1.2%
2.58_764.5205n	745.5026	2.58	282.3	280406.20	270282.68	301497.24	257447.40	285287.67	280158.57	279179.96	13474.00	4.8%
2.64_717.4699m/z	717.4699	2.64	274.8	278.42	289.59	280.71	248.41	289.16	278.36	277.44	13.79	5.0%
2.64_745.5060m/z	745.5060	2.64	347.8	125.27	109.70	139.86	125.98	114.01	125.26	123.35	9.67	7.8%
2.48_334.0963m/z	334.0963	2.48	165.6	10092.53	10053.26	10181.69	10023.62	10096.37	10089.90	10089.56	48.66	0.5%
2.45_773.5340m/z	773.5340	2.45	293.8	85382.75	82871.25	93398.73	75215.34	85665.19	85345.59	84646.48	5334.92	6.3%
2.84_509.2885m/z	509.2885	2.84	226.9	55877.45	52628.24	59471.13	53085.37	57388.27	55796.18	55707.77	2358.81	4.2%
2.82_691.4525m/z	691.4525	2.82	267.4	326.20	321.01	349.26	290.21	330.97	326.02	323.95	17.54	5.4%

2.90_672.4762n	717.4678	2.90	270.9	131.73	119.39	149.49	113.23	137.60	131.51	130.49	11.80	9.0%
2.19_618.4488m/z	618.4488	2.19	260.8	719.13	731.32	737.36	645.51	759.12	718.28	718.45	35.35	4.9%
2.22_514.8488m/z	514.8488	2.22	208.5	4308.04	4413.35	3997.69	4625.61	4127.51	4310.72	4297.15	200.06	4.7%
2.22_514.6265m/z	514.6265	2.22	210.3	8236.88	8319.08	8009.67	8511.29	8068.92	8238.55	8230.73	164.15	2.0%
2.22_514.9252m/z	514.9252	2.22	208.5	11518.56	11809.00	10940.33	12069.57	11190.44	11524.20	11508.68	371.80	3.2%
2.22_515.4955m/z	515.4955	2.22	210.3	11858.44	12051.98	11402.83	12370.84	11535.89	11862.84	11847.13	319.08	2.7%
2.22_514.4191m/z	514.4191	2.22	208.5	10112.93	10448.62	9298.95	10992.68	9508.66	10122.62	10080.74	563.95	5.6%
2.22_514.2847m/z	514.2847	2.22	208.5	1635194.42	1629218.86	1627934.31	1662746.48	1612169.05	1635118.12	1633730.20	15082.23	0.9%
2.21_184.0010m/z	184.0010	2.21	121.3	301.03	322.34	223.40	354.47	268.64	301.67	295.26	41.20	14.0%
2.12_498.8449m/z	498.8449	2.12	208.8	6797.43	6743.54	6803.14	6818.37	6803.92	6794.60	6793.50	23.57	0.3%
2.11_498.6262m/z	498.6262	2.11	207.0	13312.98	13347.38	13061.63	13680.08	13090.41	13313.35	13300.97	203.22	1.5%
2.17_206.8520m/z	206.8520	2.17	117.1	427.68	417.27	429.21	432.60	427.45	427.44	426.94	4.69	1.1%
2.17_208.8496m/z	208.8496	2.17	117.0	681.67	657.03	697.46	681.10	682.53	681.17	680.16	11.86	1.7%
2.16_434.2564m/z	434.2564	2.16	208.4	182.21	186.53	180.73	177.38	181.80	182.22	181.81	2.69	1.5%
2.37_514.5501m/z	514.5501	2.37	208.5	146.99	159.50	104.04	168.08	140.57	147.20	144.40	20.19	14.0%
2.35_706.4660m/z	706.4660	2.35	278.9	3950.27	4006.53	3951.42	3928.90	3881.47	3951.30	3944.98	36.94	0.9%
2.35_483.2728m/z	483.2728	2.35	221.9	20425.86	19698.47	21141.59	19915.87	20808.16	20403.02	20398.83	490.53	2.4%
2.30_514.5505m/z	514.5505	2.30	210.3	273.05	261.23	259.11	304.22	246.14	273.29	269.51	18.05	6.7%
2.11_498.9204m/z	498.9204	2.11	208.8	16462.74	16268.18	16450.28	16680.75	16355.33	16457.44	16445.79	126.08	0.8%
3.70_247.0373m/z	247.0373	3.70	150.3	43.71	38.37	50.72	36.61	45.97	43.62	43.16	4.68	10.8%
3.66_197.1283m/z	197.1283	3.66	147.8	0.00	4.67	0.00	5.36	5.00	4.99	3.34	2.37	71.0%
3.66_446.2565m/z	446.2565	3.66	211.8	361.66	339.17	339.28	380.24	363.10	361.28	357.45	14.42	4.0%
3.65_183.0045m/z	183.0045	3.65	124.5	6277.25	6084.03	6639.47	5828.15	6480.30	6267.57	6262.79	261.50	4.2%
4.00_663.3826m/z	663.3826	4.00	258.2	39.55	44.56	26.31	42.26	41.29	39.54	38.92	5.89	15.1%
3.98_747.7674m/z	747.7674	3.98	286.2	18999.10	18849.83	20351.13	16841.51	19190.43	18993.77	18870.96	1036.31	5.5%
4.06_665.4004m/z	665.4004	4.06	262.0	93.53	96.19	88.79	95.64	92.56	93.54	93.38	2.41	2.6%
4.05_691.4534m/z	691.4534	4.05	269.3	1370.29	1326.55	1387.10	1412.16	1328.79	1370.19	1365.85	30.42	2.2%
3.96_717.4692m/z	717.4692	3.96	272.8	702.98	650.13	749.85	698.42	690.50	702.57	699.08	29.09	4.2%
3.83_747.9328m/z	747.9328	3.83	284.2	227.56	208.57	250.29	209.09	233.45	227.20	226.03	14.37	6.4%

3.89_748.2926m/z	748.2926	3.89	286.2	112.28	96.55	134.99	72.49	118.03	111.99	107.72	19.39	18.0%
3.10_760.5184m/z	760.5184	3.10	286.0	21398.11	20426.74	23736.59	18393.83	21764.01	21379.40	21183.11	1596.59	7.5%
3.10_481.2562m/z	481.2562	3.10	218.3	1249.94	1106.66	1380.63	1112.15	1350.95	1244.72	1240.84	105.14	8.5%
3.08_747.5208m/z	747.5208	3.08	79.0	705.29	578.26	791.02	698.72	646.13	705.30	687.45	64.71	9.4%
3.13_493.2699n	448.2728	3.13	217.2	360.19	334.09	399.93	296.59	392.84	358.88	357.09	34.92	9.8%
3.54_229.0383m/z	229.0383	3.54	141.3	370.06	381.18	368.88	336.21	392.87	369.45	369.77	17.28	4.7%
3.36_422.2493n	421.2428	3.36	210.6	194.29	213.16	172.03	196.75	195.52	194.65	194.40	11.97	6.2%
1.05_246.9759m/z	246.9759	1.05	138.9	1483.96	1444.54	1508.28	1464.16	1510.94	1482.52	1482.40	23.28	1.6%
1.04_279.1802m/z	279.1802	1.04	177.7	458.98	447.02	489.32	407.54	483.19	458.21	457.38	26.71	5.8%
1.08_279.1266m/z	279.1266	1.08	165.7	932.61	906.94	992.55	861.71	948.92	931.84	929.10	39.73	4.3%
0.96_197.0268m/z	197.0268	0.96	142.9	257.25	258.41	243.85	272.20	251.52	257.27	256.75	8.52	3.3%
0.95_537.2886m/z	537.2886	0.95	239.4	4835.06	4683.21	4953.56	4839.06	4807.76	4832.52	4825.19	78.83	1.6%
0.95_183.0118m/z	183.0118	0.95	134.0	6030.18	5956.70	6023.15	6129.60	5970.66	6028.53	6023.14	55.65	0.9%
0.94_280.9363m/z	280.9363	0.94	142.5	278.83	261.55	294.25	271.65	283.00	278.49	277.96	10.01	3.6%
1.02_248.9724m/z	248.9724	1.02	138.8	136.31	138.78	122.02	149.49	130.87	136.38	135.64	8.27	6.1%
1.31_305.1778m/z	305.1778	1.31	178.5	1762.51	1727.32	1789.80	1755.40	1766.34	1761.55	1760.49	18.37	1.0%
1.31_319.1939m/z	319.1939	1.31	183.2	80.63	77.89	90.15	65.19	84.97	80.53	79.89	7.66	9.6%
1.31_323.1524m/z	323.1524	1.31	176.2	147.24	152.93	124.98	173.94	123.28	147.52	144.98	17.24	11.9%
1.29_281.1421m/z	281.1421	1.29	167.4	2173.95	2144.48	2207.45	2156.59	2176.94	2172.97	2172.07	19.50	0.9%
1.28_326.1852m/z	326.1852	1.28	190.0	231.95	238.82	219.56	251.25	209.48	232.31	230.56	13.33	5.8%
1.36_310.1803n	291.1625	1.36	175.6	2352.53	2312.90	2366.53	2381.44	2329.66	2351.87	2349.15	22.57	1.0%
1.33_392.2458m/z	392.2458	1.33	202.3	83.69	92.35	61.66	98.72	73.80	83.89	82.35	12.07	14.7%
1.33_736.4907m/z	736.4907	1.33	278.5	931.48	898.69	979.79	887.85	948.51	930.57	929.48	30.49	3.3%
1.32_341.1779m/z	341.1779	1.32	186.0	815.01	785.01	832.70	834.19	791.98	814.89	812.30	18.56	2.3%
1.26_362.2358m/z	362.2358	1.26	196.0	289.90	305.81	254.99	293.14	298.59	289.78	288.70	16.07	5.6%
1.15_771.5162m/z	771.5162	1.15	291.8	49.42	39.17	58.76	41.80	51.96	49.25	48.39	6.46	13.4%
1.25_351.2197m/z	351.2197	1.25	192.8	2543.85	2453.38	2566.41	2574.06	2548.96	2541.96	2538.10	39.66	1.6%
1.23_325.1475m/z	325.1475	1.23	186.5	181.76	183.69	184.32	167.38	192.91	181.43	181.91	7.54	4.1%
1.23_254.1390m/z	254.1390	1.23	163.4	1404.97	1355.32	1453.25	1390.27	1403.30	1404.13	1401.87	28.75	2.1%

0.83_463.3089m/z	463.3089	0.83	224.2	27.27	27.89	26.83	26.59	27.89	27.26	27.29	0.49	1.8%
0.83_492.9451m/z	492.9451	0.83	198.2	16.59	15.20	18.27	17.28	13.55	16.60	16.25	1.52	9.3%
0.83_491.3398m/z	491.3398	0.83	231.0	45.78	44.49	48.95	40.93	47.83	45.71	45.62	2.56	5.6%
0.83_287.1294m/z	287.1294	0.83	162.1	10.41	10.73	10.12	9.90	10.60	10.40	10.36	0.28	2.7%
0.83_459.4206m/z	459.4206	0.83	231.7	48.94	50.06	48.75	45.39	51.68	48.87	48.95	1.89	3.9%
0.83_377.2696m/z	377.2696	0.83	208.1	131.59	123.80	142.87	120.38	135.62	131.40	130.94	7.39	5.6%
0.83_489.2658m/z	489.2658	0.83	240.4	1633.83	1592.03	1651.83	1652.05	1623.31	1633.06	1631.02	20.28	1.2%
0.82_533.2918m/z	533.2918	0.82	247.1	0.00	4.35	3.27	4.20	0.00	3.85	2.61	1.88	71.9%
0.82_518.4104n	553.3723	0.82	244.8	204.59	189.10	221.15	195.30	205.73	204.36	203.37	9.94	4.9%
0.84_293.9579m/z	293.9579	0.84	145.3	408.32	398.59	414.17	400.31	419.93	407.75	408.18	7.39	1.8%
0.84_459.2930m/z	459.2930	0.84	229.9	622.79	589.67	655.58	606.09	628.69	622.17	620.83	20.26	3.3%
0.84_301.1110m/z	301.1110	0.84	175.2	571.40	551.70	596.44	548.23	584.27	570.75	570.46	16.90	3.0%
0.84_669.2877m/z	669.2877	0.84	285.4	1758.42	1648.99	1866.59	1721.16	1751.84	1757.03	1750.67	64.23	3.7%
0.84_475.2888m/z	475.2888	0.84	235.1	3674.12	3521.31	3898.59	3527.75	3671.05	3671.82	3660.77	125.24	3.4%
0.84_473.4804m/z	473.4804	0.84	235.1	1471.90	1412.56	1541.65	1421.66	1493.94	1470.42	1468.69	43.47	3.0%
0.84_473.2828m/z	473.2828	0.84	235.1	98680.30	95954.85	101282.34	97735.04	98886.02	98618.20	98526.13	1580.92	1.6%
0.84_473.8976m/z	473.8976	0.84	233.3	188.70	177.42	196.29	195.63	178.34	188.72	187.52	7.44	4.0%
0.84_473.6108m/z	473.6108	0.84	233.3	259.25	247.17	273.10	262.14	243.43	259.32	257.40	9.80	3.8%
0.84_534.2583m/z	534.2583	0.84	243.3	60.54	58.79	61.09	61.16	60.67	60.50	60.46	0.79	1.3%
0.84_310.1290m/z	310.1290	0.84	169.7	58.86	58.76	59.61	56.69	60.51	58.80	58.87	1.15	2.0%
0.84_412.2099m/z	412.2099	0.84	212.6	33.06	31.38	35.07	31.31	34.00	33.01	32.97	1.34	4.1%
0.84_280.0972n	325.1030	0.84	169.3	129.18	130.69	125.69	133.99	125.08	129.24	128.98	3.00	2.3%
0.84_439.2846m/z	439.2846	0.84	226.6	115.46	121.38	109.11	113.99	117.60	115.47	115.50	3.70	3.2%
0.84_661.4389m/z	661.4389	0.84	275.7	1047.87	1010.12	1082.19	1041.36	1043.76	1047.26	1045.43	20.93	2.0%
0.84_439.3035m/z	439.3035	0.84	219.2	1743.52	1703.79	1815.16	1681.67	1754.48	1742.48	1740.18	42.01	2.4%
0.84_535.3058m/z	535.3058	0.84	250.8	1498.94	1412.78	1609.10	1428.42	1503.74	1497.64	1491.77	63.57	4.3%
0.82_519.3690m/z	519.3690	0.82	241.7	155.82	145.60	165.56	152.48	155.45	155.69	155.10	5.88	3.8%
0.82_485.4356m/z	485.4356	0.82	238.6	135.38	138.44	130.32	143.70	125.39	135.57	134.80	5.80	4.3%
0.82_603.3666m/z	603.3666	0.82	251.5	217.13	215.34	219.18	219.61	211.93	217.14	216.72	2.57	1.2%

0.79_605.3768m/z	605.3768	0.79	268.8	21.78	21.81	21.85	22.49	20.69	21.79	21.73	0.53	2.4%
0.79_709.4496m/z	709.4496	0.79	292.8	20.33	20.53	20.72	18.10	21.85	20.29	20.30	1.12	5.5%
0.82_555.3897m/z	555.3897	0.82	244.7	570.82	553.25	597.37	556.09	567.27	570.57	569.23	14.31	2.5%
0.82_666.0588m/z	666.0588	0.82	260.0	468.09	460.49	476.20	466.51	465.81	467.93	467.50	4.64	1.0%
0.81_722.1223m/z	722.1223	0.81	280.6	105.45	104.99	106.15	102.79	108.01	105.35	105.46	1.55	1.5%
0.80_387.7122m/z	387.7122	0.80	204.2	143.34	140.08	147.98	138.34	146.19	143.21	143.19	3.30	2.3%
0.80_387.4525m/z	387.4525	0.80	202.4	166.54	164.40	168.65	165.36	167.05	166.46	166.41	1.33	0.8%
0.80_383.2222m/z	383.2222	0.80	213.4	41.15	39.01	42.18	41.56	41.16	41.11	41.03	0.98	2.4%
0.80_619.3629m/z	619.3629	0.80	258.9	181.43	180.81	184.54	177.51	182.59	181.37	181.38	2.11	1.2%
0.80_387.6458m/z	387.6458	0.80	204.2	72.62	71.62	73.05	74.26	70.64	72.62	72.47	1.13	1.6%
0.80_388.1615m/z	388.1615	0.80	87.9	42.33	38.98	44.20	40.90	44.72	42.21	42.22	1.93	4.6%
0.80_389.1614m/z	389.1614	0.80	204.2	885.36	867.95	904.56	872.02	892.37	884.75	884.50	12.21	1.4%
0.80_387.3343m/z	387.3343	0.80	202.4	598.87	579.68	616.30	594.42	598.83	598.51	597.77	10.68	1.8%
0.80_387.1563m/z	387.1563	0.80	204.2	35074.24	34465.35	35682.59	34807.07	35138.47	35055.94	35037.28	367.08	1.0%
0.84_518.2685m/z	518.2685	0.84	239.8	21.63	21.74	23.81	11.47	24.70	21.56	20.82	4.35	20.9%
0.90_573.3290m/z	573.3290	0.90	240.6	220.57	215.98	213.46	227.67	224.38	220.10	220.36	4.77	2.2%
0.90_553.2812m/z	553.2812	0.90	241.0	291.24	295.59	269.07	320.48	269.06	291.57	289.50	17.51	6.0%
0.89_400.2262m/z	400.2262	0.89	202.1	10.42	7.68	13.20	6.73	0.00	10.41	8.07	4.17	51.7%
0.89_469.2827m/z	469.2827	0.89	222.2	2274.02	2202.78	2351.59	2257.24	2252.98	2273.08	2268.62	44.05	1.9%
0.89_381.2315m/z	381.2315	0.89	199.0	11116.76	10887.39	11389.52	10951.43	11163.69	11110.12	11103.15	160.98	1.4%
0.92_522.2950m/z	522.2950	0.92	237.8	249.23	222.03	287.44	205.12	264.35	248.72	246.15	26.83	10.9%
0.86_453.2877m/z	453.2877	0.86	220.7	201.66	186.49	215.13	193.08	207.88	201.30	200.92	9.32	4.6%
0.86_368.9758m/z	368.9758	0.86	138.6	761.76	757.53	756.66	765.55	766.24	761.34	761.51	3.61	0.5%
0.86_285.1160m/z	285.1160	0.86	175.8	1142.12	1112.37	1153.21	1162.97	1126.43	1141.75	1139.81	16.62	1.5%
0.86_294.1343m/z	294.1343	0.86	170.3	863.51	831.31	900.71	842.21	869.09	862.84	861.61	21.93	2.5%
0.86_291.9611m/z	291.9611	0.86	145.4	1717.71	1701.55	1702.02	1734.74	1727.76	1716.70	1716.75	12.21	0.7%
0.86_318.9795m/z	318.9795	0.86	130.3	2194.43	2155.86	2213.03	2200.47	2196.71	2193.21	2192.28	17.56	0.8%
0.86_485.2808m/z	485.2808	0.86	231.1	7107.45	6881.77	7355.21	7042.53	7053.79	7104.24	7090.83	140.10	2.0%
0.85_341.1833m/z	341.1833	0.85	189.5	2353.48	2269.27	2427.06	2308.48	2390.18	2351.05	2349.92	51.33	2.2%

0.85_421.2264m/z	421.2264	0.85	212.4	14207.31	13771.06	14628.61	14069.37	14209.87	14198.65	14180.81	252.56	1.8%
0.84_473.5380m/z	473.5380	0.84	235.1	52.63	57.11	46.22	50.03	56.56	52.56	52.52	3.73	7.1%
0.87_280.1188m/z	280.1188	0.87	165.7	2053.77	2014.18	2092.59	2046.22	2046.18	2052.86	2050.97	22.88	1.1%
0.89_465.3034m/z	465.3034	0.89	242.9	1162.62	1141.57	1190.38	1141.18	1170.58	1161.91	1161.37	16.97	1.5%
0.89_653.3012m/z	653.3012	0.89	269.9	21098.37	20621.25	21493.89	21124.61	20957.17	21089.81	21064.18	257.48	1.2%
0.87_321.3734m/z	321.3734	0.87	184.9	133.73	129.99	146.27	109.55	142.69	133.50	132.62	11.75	8.9%
0.87_327.1652m/z	327.1652	0.87	186.5	367.67	358.15	377.45	356.84	376.91	367.23	367.38	8.05	2.2%
0.87_365.2353m/z	365.2353	0.87	197.7	1136.24	1100.49	1181.37	1101.72	1150.71	1135.23	1134.30	27.97	2.5%
0.87_349.2409m/z	349.2409	0.87	194.6	1908.13	1849.46	1973.74	1878.35	1909.95	1906.94	1904.43	37.76	2.0%
0.88_409.2611m/z	409.2611	0.88	207.2	528.88	502.26	552.43	520.09	533.15	528.38	527.53	14.99	2.8%
1.36_379.2148m/z	379.2148	1.36	195.5	1455.38	1391.34	1485.82	1486.43	1427.09	1454.86	1450.16	33.24	2.3%
1.36_651.3855m/z	651.3855	1.36	258.3	1076.31	1031.52	1088.43	1091.64	1079.65	1075.44	1073.83	19.84	1.8%
1.79_317.0903m/z	317.0903	1.79	164.4	1627.24	1639.72	1578.09	1687.07	1593.54	1627.47	1625.52	34.80	2.1%
1.79_496.2736m/z	496.2736	1.79	208.9	39407.96	37353.48	39722.87	41086.85	38292.23	39397.23	39210.10	1165.39	3.0%
1.86_213.9631m/z	213.9631	1.86	138.7	1224.44	1213.87	1213.67	1266.09	1189.79	1224.61	1222.08	22.84	1.9%
1.69_249.0314m/z	249.0314	1.69	153.5	673.37	656.44	657.77	691.90	676.83	672.86	671.53	12.01	1.8%
1.76_492.2405m/z	492.2405	1.76	218.1	3205.31	3119.35	3269.90	3178.86	3231.30	3202.85	3201.26	46.27	1.4%
1.74_167.0089m/z	167.0089	1.74	122.3	103.50	104.25	104.16	99.74	106.07	103.45	103.53	1.90	1.8%
1.74_236.1073m/z	236.1073	1.74	147.5	1270.22	1226.44	1294.41	1264.56	1283.44	1269.03	1268.02	21.14	1.7%
2.10_498.4907m/z	498.4907	2.10	207.1	59142.89	58569.85	58549.29	60898.19	57999.69	59139.32	59049.87	914.40	1.5%
2.10_498.2900m/z	498.2900	2.10	208.9	2219485.56	2197857.98	2209318.77	2273893.80	2176507.55	2219371.61	2216072.55	29745.03	1.3%
1.91_581.2968m/z	581.2968	1.91	238.6	411.08	413.76	382.85	430.72	411.43	410.91	410.13	14.05	3.4%
1.90_541.2934m/z	541.2934	1.90	220.8	1106.46	1125.25	974.19	1251.56	1010.27	1107.80	1095.92	88.97	8.1%
1.96_555.2841m/z	555.2841	1.96	233.5	141574.59	140662.96	140709.69	144447.43	139527.26	141557.57	141413.25	1520.31	1.1%
1.46_513.2877m/z	513.2877	1.46	219.5	1137.65	1121.70	1159.63	1140.01	1116.03	1137.56	1135.43	14.03	1.2%
1.46_295.1216m/z	295.1216	1.46	166.9	2713.22	2693.87	2672.19	2779.29	2690.35	2712.55	2710.24	33.89	1.3%
1.45_476.2447m/z	476.2447	1.45	216.6	521.59	502.50	536.91	537.71	494.32	521.70	519.12	16.16	3.1%
1.45_449.1493m/z	449.1493	1.45	217.2	20966.32	20395.29	21222.48	21005.95	21075.35	20951.34	20936.12	258.17	1.2%
1.49_367.2142m/z	367.2142	1.49	195.8	518.33	485.92	551.98	500.76	522.61	517.83	516.24	20.35	3.9%

1.49_351.1841m/z	351.1841	1.49	189.2	2298.00	2267.97	2324.36	2279.14	2311.22	2296.78	2296.25	18.75	0.8%
1.49_447.1342m/z	447.1342	1.49	211.8	59839.79	58504.43	60531.55	60034.01	59857.43	59806.69	59762.32	614.39	1.0%
1.47_377.0683m/z	377.0683	1.47	176.4	396.69	397.27	386.24	419.34	377.94	396.86	395.72	12.70	3.2%
1.47_321.1723m/z	321.1723	1.47	176.2	789.06	770.36	800.03	790.72	788.84	788.62	787.94	8.81	1.1%
1.39_307.1944m/z	307.1944	1.39	181.9	4446.24	4346.16	4536.07	4416.61	4452.62	4443.82	4440.25	55.97	1.3%
1.38_269.1301m/z	269.1301	1.38	176.4	125.95	113.70	137.30	121.16	125.58	125.81	124.92	7.02	5.6%
1.38_499.3094m/z	499.3094	1.38	221.6	6592.89	6439.34	6658.41	6637.05	6587.78	6589.21	6584.11	70.00	1.1%
1.37_327.1627m/z	327.1627	1.37	183.0	1132.51	1110.90	1130.39	1174.92	1094.83	1132.63	1129.36	24.60	2.2%
1.39_493.1942m/z	493.1942	1.39	210.8	421.46	382.05	444.06	446.53	385.35	421.58	416.84	25.39	6.1%
1.42_211.0175m/z	211.0175	1.42	137.3	285.86	249.22	316.21	275.47	283.91	285.54	282.70	19.65	6.9%
1.42_385.1773m/z	385.1773	1.42	204.3	1690.44	1647.14	1690.07	1746.95	1651.69	1690.19	1686.08	32.80	1.9%
1.64_514.2660m/z	514.2660	1.64	214.0	8797.12	8690.93	8721.99	9046.67	8644.51	8796.11	8782.89	129.92	1.5%
1.62_278.8476m/z	278.8476	1.62	128.3	346.24	354.30	295.97	376.20	343.68	346.16	343.76	24.01	7.0%
1.62_515.3045m/z	515.3045	1.62	215.8	201407.55	197335.60	202697.41	204518.37	199156.75	201345.27	201076.83	2321.47	1.2%
1.68_204.9728m/z	204.9728	1.68	137.6	818.04	779.22	831.61	841.69	799.02	817.77	814.56	20.57	2.5%
1.67_152.9954m/z	152.9954	1.67	121.7	225.56	218.36	231.48	227.20	221.64	225.50	224.96	4.14	1.8%
1.67_187.0063m/z	187.0063	1.67	132.2	928.42	897.67	924.91	954.16	921.34	927.91	925.74	16.47	1.8%
1.53_372.8222m/z	372.8222	1.53	133.7	434.18	422.87	385.90	479.04	420.86	434.15	429.50	27.44	6.4%
1.52_294.1366m/z	294.1366	1.52	168.6	108.92	105.63	100.94	117.52	106.44	108.90	108.06	5.00	4.6%
1.52_480.2781m/z	480.2781	1.52	214.7	7130.76	6975.85	7176.12	7172.33	7152.36	7126.37	7122.30	68.12	1.0%
1.51_251.0493m/z	251.0493	1.51	155.1	372.40	368.01	362.46	398.43	350.29	372.62	370.70	14.53	3.9%
1.58_361.1159m/z	361.1159	1.58	176.8	1194.82	1165.06	1155.69	1277.91	1148.02	1194.96	1189.41	43.46	3.7%
1.57_508.2358m/z	508.2358	1.57	217.7	116.05	122.81	106.14	121.44	113.31	116.16	115.99	5.48	4.7%
1.57_529.3172m/z	529.3172	1.57	221.0	1367.95	1371.84	1376.10	1331.98	1393.90	1367.01	1368.13	18.48	1.4%
1.57_403.1888m/z	403.1888	1.57	207.4	23140.26	22631.47	23121.03	23747.24	22788.69	23134.30	23093.83	350.80	1.5%
1.54_307.1571m/z	307.1571	1.54	176.7	878.16	879.43	867.28	879.71	886.01	877.74	878.06	5.54	0.6%
6.36_204.0659m/z	204.0659	6.36	147.4	328.05	328.77	331.99	318.69	332.53	327.88	327.99	4.54	1.4%
6.35_725.3823m/z	725.3823	6.35	249.6	93.90	93.20	90.91	97.74	92.99	93.87	93.77	2.04	2.2%
6.33_571.3065m/z	571.3065	6.33	223.9	102.12	100.74	107.30	98.02	100.11	102.12	101.74	2.85	2.8%

6.33_537.3003m/z	537.3003	6.33	226.4	103.98	106.80	104.91	99.05	104.71	104.00	103.91	2.37	2.3%
6.33_412.2189m/z	412.2189	6.33	196.4	232.67	226.37	228.25	246.33	223.98	232.70	231.72	7.26	3.1%
6.33_314.1139m/z	314.1139	6.33	174.7	597.63	596.46	595.52	595.84	602.75	597.30	597.58	2.42	0.4%
6.32_693.3208m/z	693.3208	6.32	246.3	2324.75	2315.27	2310.64	2350.64	2315.52	2324.04	2323.48	13.13	0.6%
6.31_567.3097m/z	567.3097	6.31	231.4	7.64	7.68	7.60	6.50	8.48	7.60	7.58	0.58	7.6%
6.37_467.1895m/z	467.1895	6.37	198.7	2607.13	2606.32	2593.95	2640.46	2575.52	2606.92	2605.05	19.40	0.7%
6.41_413.2353m/z	413.2353	6.41	196.4	564.13	566.37	564.93	559.96	565.28	563.99	564.11	2.02	0.4%
6.40_650.2716m/z	650.2716	6.40	248.8	92.89	93.35	96.76	84.72	96.19	92.82	92.79	3.93	4.2%
6.39_261.1241m/z	261.1241	6.39	163.1	803.25	799.94	804.98	805.36	800.00	803.04	802.76	2.14	0.3%
6.38_413.2334m/z	413.2334	6.38	196.4	115.98	115.67	120.14	112.93	112.20	116.02	115.49	2.57	2.2%
6.38_284.1248m/z	284.1248	6.38	167.3	3062.09	3070.23	3037.57	3084.65	3050.52	3061.43	3061.08	14.75	0.5%
6.22_537.3030m/z	537.3030	6.22	226.4	60.99	52.83	63.55	65.17	57.23	60.99	60.13	4.09	6.8%
6.21_583.2490m/z	583.2490	6.21	233.0	40.52	40.92	40.47	37.07	43.86	40.38	40.54	1.97	4.9%
6.21_328.1503m/z	328.1503	6.21	184.7	387.02	391.91	390.00	376.62	389.30	386.96	386.97	4.93	1.3%
6.20_274.2437m/z	274.2437	6.20	157.5	23.49	22.83	23.89	22.63	24.82	23.43	23.51	0.72	3.1%
6.19_286.1354m/z	286.1354	6.19	168.9	21.69	22.18	19.90	23.29	21.04	21.70	21.64	1.03	4.8%
6.24_580.2355m/z	580.2355	6.24	223.8	119.32	120.98	115.58	117.86	124.06	119.12	119.49	2.62	2.2%
6.30_207.0865m/z	207.0865	6.30	144.0	76.54	74.81	82.91	67.30	78.53	76.48	76.10	4.69	6.2%
6.28_413.2360m/z	413.2360	6.28	196.4	206.46	210.77	199.52	210.39	205.28	206.47	206.48	3.73	1.8%
6.28_194.9962m/z	194.9962	6.28	128.5	374.74	368.61	390.21	352.59	385.35	374.32	374.30	12.10	3.2%
6.28_511.2868m/z	511.2868	6.28	219.5	38.18	36.34	40.81	35.00	40.11	38.09	38.09	2.00	5.3%
6.27_355.1619m/z	355.1619	6.27	180.4	2118.20	2119.22	2114.64	2125.18	2108.28	2117.78	2117.22	5.08	0.2%
6.26_399.0993m/z	399.0993	6.26	184.5	490.08	490.42	484.00	499.56	484.38	490.04	489.75	5.14	1.1%
6.19_721.3491m/z	721.3491	6.19	255.4	365.21	356.03	364.86	384.60	345.05	365.36	363.52	11.90	3.3%
6.62_385.1705m/z	385.1705	6.62	188.3	59.13	58.84	60.80	56.02	60.75	59.07	59.10	1.59	2.7%
6.58_624.3344m/z	624.3344	6.58	237.9	896.44	901.62	894.16	895.35	894.14	896.31	896.34	2.53	0.3%
6.57_610.2844m/z	610.2844	6.57	228.8	50.77	46.61	57.41	43.86	51.41	50.71	50.13	4.22	8.4%
6.56_679.3016m/z	679.3016	6.56	250.3	11.04	11.86	9.30	12.17	10.55	11.06	11.00	0.93	8.5%
6.63_580.2355m/z	580.2355	6.63	218.3	542.00	545.66	532.94	559.33	524.41	542.23	541.09	10.80	2.0%

6.67_601.2970m/z	601.2970	6.67	234.5	827.74	822.24	818.02	848.75	816.44	827.62	826.80	10.71	1.3%
6.66_665.3604m/z	665.3604	6.66	246.7	569.36	570.78	549.00	598.98	551.39	569.53	568.17	16.37	2.9%
6.65_316.1862m/z	316.1862	6.65	183.3	35.88	34.02	37.55	35.75	35.34	35.87	35.74	1.04	2.9%
6.65_594.3246m/z	594.3246	6.65	238.4	1600.53	1578.46	1594.60	1644.64	1566.64	1600.44	1597.55	24.37	1.5%
6.64_502.1968m/z	502.1968	6.64	212.4	319.82	323.41	311.02	325.23	320.48	319.68	319.94	4.47	1.4%
6.56_307.9499n	288.9320	6.56	150.4	165.50	160.82	164.69	168.32	166.55	165.35	165.21	2.28	1.4%
6.56_227.1396m/z	227.1396	6.56	156.2	2700.05	2673.69	2675.69	2748.89	2688.21	2699.06	2697.60	25.10	0.9%
6.47_508.2362m/z	508.2362	6.47	215.9	103.52	104.33	104.61	101.26	104.09	103.50	103.55	1.10	1.1%
6.46_636.2968m/z	636.2968	6.46	235.8	550.26	553.16	538.62	564.95	542.16	550.28	549.91	8.41	1.5%
6.45_794.3688m/z	794.3688	6.45	262.1	1896.10	1921.19	1865.84	1919.35	1877.77	1896.29	1896.09	20.09	1.1%
6.48_644.2940m/z	644.2940	6.48	239.4	7.07	6.40	8.13	5.86	7.28	7.06	6.97	0.71	10.2%
6.48_454.2595m/z	454.2595	6.48	209.8	15.76	15.28	16.81	15.05	15.44	15.76	15.68	0.56	3.6%
6.49_296.0996m/z	296.0996	6.49	165.1	326.68	334.37	314.59	337.95	318.43	326.84	326.48	8.16	2.5%
6.55_385.1694m/z	385.1694	6.55	188.3	22.51	23.27	21.85	22.69	22.17	22.52	22.50	0.44	1.9%
6.55_272.1232m/z	272.1232	6.55	162.6	99.60	102.16	99.00	95.58	101.85	99.55	99.62	2.17	2.2%
6.55_565.2618m/z	565.2618	6.55	224.0	1670.91	1663.39	1654.86	1706.98	1648.62	1670.70	1669.24	18.68	1.1%
6.54_610.3189m/z	610.3189	6.54	226.9	3063.99	3077.18	2997.58	3135.66	3037.51	3063.58	3062.58	41.65	1.4%
6.53_626.3500m/z	626.3500	6.53	245.4	46.26	48.15	43.36	46.46	47.46	46.24	46.32	1.50	3.2%
6.53_225.0873m/z	225.0873	6.53	151.3	1677.58	1667.34	1676.68	1688.72	1671.72	1677.04	1676.51	6.55	0.4%
6.52_555.3128m/z	555.3128	6.52	229.7	772.77	769.09	755.44	794.60	768.14	772.52	772.10	11.61	1.5%
6.50_681.3563m/z	681.3563	6.50	248.3	111.44	116.05	105.11	114.00	110.74	111.48	111.47	3.37	3.0%
6.50_610.2816m/z	610.2816	6.50	232.5	87.17	93.24	82.27	86.42	86.55	87.28	87.16	3.21	3.7%
6.19_585.2676m/z	585.2676	6.19	238.5	305.96	315.58	303.05	295.59	310.33	305.96	306.08	6.16	2.0%
5.94_328.1500m/z	328.1500	5.94	179.5	476.02	464.01	480.86	483.47	470.86	475.84	475.18	6.40	1.3%
5.93_795.5581m/z	795.5581	5.93	297.5	2.18	2.02	2.43	0.00	0.00	2.19	1.47	1.05	71.2%
5.93_465.1448m/z	465.1448	5.93	216.8	1117.73	1089.84	1139.07	1129.47	1096.91	1117.51	1115.09	17.14	1.5%
5.92_749.4437m/z	749.4437	5.92	278.3	3429.28	3407.37	3422.86	3454.72	3421.30	3428.09	3427.27	14.20	0.4%
5.91_298.1403m/z	298.1403	5.91	171.9	2286.20	2259.40	2314.03	2282.27	2275.92	2285.45	2283.88	16.24	0.7%
5.90_440.2505m/z	440.2505	5.90	204.7	1383.03	1359.15	1399.45	1379.40	1387.87	1382.18	1381.85	12.03	0.9%

5.95_491.2145m/z	491.2145	5.95	209.0	4049.29	4010.03	4072.87	4062.25	4033.26	4047.88	4045.93	20.23	0.5%
5.95_451.2371m/z	451.2371	5.95	166.3	1221.09	1207.56	1214.30	1256.84	1192.16	1221.10	1218.84	19.64	1.6%
5.95_263.1386m/z	263.1386	5.95	164.7	42.71	43.21	42.75	42.90	40.73	42.73	42.50	0.81	1.9%
5.98_332.1224m/z	332.1224	5.98	179.3	20.48	20.12	18.73	23.93	16.24	20.54	20.01	2.30	11.5%
6.02_490.2617n	525.2311	6.02	212.0	6079.73	6061.94	6049.93	6169.24	6006.56	6079.06	6074.41	48.99	0.8%
5.98_711.3311m/z	711.3311	5.98	253.6	3168.70	3143.09	3179.92	3211.48	3114.68	3168.55	3164.40	30.05	0.9%
5.99_407.7344n	406.7271	5.99	158.8	1557.53	1536.73	1590.43	1535.23	1558.63	1556.83	1555.90	18.25	1.2%
5.97_451.7426n	450.7353	5.97	166.3	7231.44	7169.63	7348.77	7174.21	7187.04	7229.69	7223.46	61.18	0.8%
5.96_633.3173m/z	633.3173	5.96	247.2	1711.05	1684.18	1712.49	1748.72	1681.29	1710.81	1708.09	22.27	1.3%
5.83_266.0888m/z	266.0888	5.83	156.2	729.17	695.50	756.55	734.49	716.01	728.90	726.77	18.48	2.5%
5.79_357.1410m/z	357.1410	5.79	185.6	2245.27	2150.25	2357.81	2172.80	2266.39	2243.18	2239.28	67.31	3.0%
5.86_530.2776m/z	530.2776	5.86	211.9	790.38	769.71	802.27	791.55	792.16	789.83	789.32	9.72	1.2%
5.86_381.1772m/z	381.1772	5.86	188.4	2467.56	2452.12	2476.24	2485.69	2442.59	2467.16	2465.23	14.34	0.6%
5.85_454.2549m/z	454.2549	5.85	217.1	116.57	112.20	117.88	124.25	107.90	116.62	115.90	5.04	4.3%
6.13_612.2745n	611.2838	6.13	243.7	1750.86	1739.88	1764.39	1744.93	1748.30	1750.27	1749.77	7.51	0.4%
6.12_286.1400m/z	286.1400	6.12	167.2	23.36	19.73	25.58	24.15	21.87	23.35	23.01	1.83	8.0%
6.12_417.2127m/z	417.2127	6.12	192.8	524.83	514.17	524.40	538.33	516.84	524.68	523.87	7.69	1.5%
6.12_261.0872m/z	261.0872	6.12	159.7	806.52	793.07	825.33	793.02	809.66	806.09	805.61	10.97	1.4%
6.11_765.4389m/z	765.4389	6.11	280.0	4325.68	4310.25	4297.68	4404.60	4265.29	4325.35	4321.48	42.41	1.0%
6.18_274.0932m/z	274.0932	6.18	157.5	10082.77	10011.34	10106.90	10151.16	10012.89	10080.27	10074.22	49.69	0.5%
6.18_479.1953m/z	479.1953	6.18	162.4	229.46	229.74	227.17	230.70	229.97	229.38	229.40	1.09	0.5%
6.17_580.2344m/z	580.2344	6.17	220.1	60.40	63.22	53.29	67.29	55.29	60.49	60.00	4.68	7.8%
6.16_305.1366m/z	305.1366	6.16	140.2	36.35	35.03	36.91	38.31	34.13	36.36	36.18	1.33	3.7%
6.16_383.2292m/z	383.2292	6.16	191.9	1072.63	1060.19	1069.91	1095.88	1055.45	1072.48	1071.09	12.80	1.2%
6.16_548.2360m/z	548.2360	6.16	217.0	4016.71	3996.08	3988.60	4090.39	3969.63	4016.07	4012.91	38.24	1.0%
6.15_482.2244m/z	482.2244	6.15	212.8	8.24	8.17	7.98	7.80	8.95	8.21	8.23	0.36	4.4%
6.10_521.2368m/z	521.2368	6.10	210.2	41.56	37.20	46.62	38.77	40.64	41.53	41.05	2.94	7.2%
6.03_779.4533m/z	779.4533	6.03	283.8	22.74	20.52	24.96	20.29	24.50	22.66	22.61	1.77	7.8%
6.03_200.0014m/z	200.0014	6.03	133.0	232.46	225.53	244.10	219.77	238.03	232.19	232.01	7.90	3.4%

6.02_592.2955m/z	592.2955	6.02	230.9	372.14	360.74	388.72	351.51	385.33	371.60	371.67	12.93	3.5%
6.02_580.2326m/z	580.2326	6.02	225.6	53.03	43.81	60.69	46.00	57.66	52.79	52.33	5.94	11.4%
6.05_438.1992m/z	438.1992	6.05	195.8	11346.10	11289.12	11313.60	11497.87	11225.42	11344.28	11336.06	82.97	0.7%
6.08_229.1553m/z	229.1553	6.08	159.4	1651.39	1584.46	1724.75	1614.69	1659.30	1650.12	1647.45	43.16	2.6%
6.07_126.9051m/z	126.9051	6.07	103.8	4859.09	4912.56	4656.94	5086.45	4742.46	4860.34	4852.97	134.66	2.8%
6.07_706.4662m/z	706.4662	6.07	280.9	205.51	207.32	212.47	194.29	205.62	205.53	205.12	5.43	2.6%
6.07_559.2154m/z	559.2154	6.07	218.6	3658.07	3640.86	3649.45	3716.28	3600.81	3657.86	3653.89	33.99	0.9%
6.05_521.2352m/z	521.2352	6.05	213.8	43.47	41.90	43.24	42.96	45.27	43.38	43.37	1.00	2.3%
7.25_810.3943m/z	810.3943	7.25	263.8	28.96	30.64	27.79	27.22	29.96	28.96	28.92	1.17	4.0%
7.21_551.2812m/z	551.2812	7.21	231.7	130.04	126.58	127.65	144.45	116.28	130.13	129.19	8.27	6.4%
7.20_341.1818m/z	341.1818	7.20	179.1	395.28	404.59	340.32	424.78	394.39	395.18	392.42	25.59	6.5%
7.28_869.4356m/z	869.4356	7.28	284.6	1783.13	1757.69	1792.39	1823.50	1735.75	1783.24	1779.28	27.44	1.5%
7.28_610.3203m/z	610.3203	7.28	234.4	5644.91	5603.42	5595.04	5801.32	5529.06	5644.92	5636.44	83.33	1.5%
7.27_341.1823m/z	341.1823	7.27	179.1	1176.01	1167.68	1188.57	1195.52	1131.58	1176.41	1172.63	20.47	1.7%
7.26_486.1661m/z	486.1661	7.26	205.5	96.50	93.41	98.78	98.31	93.28	96.50	96.13	2.15	2.2%
7.26_569.2916m/z	569.2916	7.26	223.9	547.03	538.48	529.75	589.07	513.51	547.33	544.19	23.15	4.3%
7.14_620.2661m/z	620.2661	7.14	228.6	513.97	506.68	517.57	523.06	502.70	513.96	512.99	6.71	1.3%
7.14_277.1191m/z	277.1191	7.14	164.1	2414.86	2385.64	2284.81	2562.73	2377.19	2414.28	2406.59	82.30	3.4%
7.14_424.2190m/z	424.2190	7.14	201.5	124.03	120.37	123.03	124.23	126.86	123.88	123.73	1.91	1.5%
7.14_355.1973m/z	355.1973	7.14	185.6	581.22	557.35	582.26	607.61	563.88	581.13	578.91	16.00	2.8%
7.12_682.3434m/z	682.3434	7.12	246.4	41.17	43.52	40.70	35.45	44.55	41.13	41.08	2.88	7.0%
7.10_810.3979m/z	810.3979	7.10	271.6	373.28	373.49	353.25	392.81	368.98	373.21	372.50	11.53	3.1%
7.10_584.3047m/z	584.3047	7.10	225.5	5278.23	5142.78	5213.30	5554.99	5089.58	5278.57	5259.58	148.69	2.8%
7.19_199.0721m/z	199.0721	7.19	146.1	757.39	752.08	765.39	746.89	763.69	757.01	757.08	6.35	0.8%
7.15_455.1243m/z	455.1243	7.15	199.0	1271.86	1273.43	1269.94	1278.80	1260.83	1271.73	1271.10	5.36	0.4%
7.30_282.1086m/z	282.1086	7.30	163.9	752.99	758.95	752.86	735.79	767.02	752.52	753.36	9.38	1.2%
7.41_497.2001m/z	497.2001	7.41	207.1	2419.54	2442.53	2392.84	2460.62	2367.90	2420.11	2417.26	30.46	1.3%
7.43_610.2641n	609.2625	7.43	230.7	577.82	589.00	570.89	577.11	573.18	577.91	577.65	5.70	1.0%
7.40_598.7630m/z	598.7630	7.40	184.2	1558.88	1553.45	1542.82	1603.98	1520.50	1559.05	1556.45	25.03	1.6%

7.39_585.2896m/z	585.2896	7.39	231.1	251.35	251.90	245.08	257.45	250.33	251.28	251.23	3.60	1.4%
7.45_229.0825m/z	229.0825	7.45	151.1	507.62	516.57	501.43	502.69	511.23	507.51	507.84	5.09	1.0%
7.45_945.4313m/z	945.4313	7.45	291.6	3399.99	3428.10	3348.04	3460.93	3349.19	3400.25	3397.75	40.32	1.2%
7.32_742.1671m/z	742.1671	7.32	249.4	120.78	117.91	128.33	111.75	121.56	120.70	120.17	4.92	4.1%
7.31_456.2453m/z	456.2453	7.31	206.1	1822.86	1795.07	1832.76	1861.99	1779.67	1822.83	1819.20	26.41	1.5%
7.35_636.3674n	671.3368	7.35	239.0	3695.03	3690.85	3598.06	3870.50	3573.33	3696.06	3687.30	95.45	2.6%
7.30_284.1609m/z	284.1609	7.30	170.7	1107.35	1089.68	1111.89	1136.19	1077.16	1107.40	1104.95	18.44	1.7%
7.33_329.1820m/z	329.1820	7.33	176.0	929.53	913.55	925.68	960.14	906.25	929.51	927.44	16.95	1.8%
7.36_741.3782m/z	741.3782	7.36	258.9	3753.70	3742.49	3719.65	3831.50	3699.31	3753.45	3750.02	41.26	1.1%
7.35_533.2330m/z	533.2330	7.35	215.4	215.00	214.55	205.28	231.09	204.04	215.10	214.18	8.85	4.1%
7.35_399.2152m/z	399.2152	7.35	196.8	365.18	363.15	354.85	387.26	348.31	365.34	364.02	12.07	3.3%
7.35_318.6302n	636.2552	7.35	247.1	4340.95	4420.38	4246.26	4386.20	4310.64	4341.26	4340.95	55.14	1.3%
7.33_438.1975m/z	438.1975	7.33	202.9	214.33	211.15	214.53	224.72	202.61	214.42	213.63	6.49	3.0%
6.85_454.1925m/z	454.1925	6.85	202.6	400.22	397.79	385.21	419.82	393.31	400.17	399.42	10.48	2.6%
6.84_452.7319n	451.7246	6.84	167.9	1704.44	1736.68	1655.05	1755.23	1663.32	1705.19	1703.32	35.92	2.1%
6.81_559.2140m/z	559.2140	6.81	216.8	1249.39	1252.16	1241.90	1264.34	1234.24	1249.35	1248.56	9.24	0.7%
6.81_438.2351m/z	438.2351	6.81	206.5	3062.39	3027.35	3049.68	3125.17	3024.72	3061.73	3058.51	33.32	1.1%
6.87_426.2351m/z	426.2351	6.87	199.6	2529.86	2457.55	2518.87	2640.63	2455.04	2529.70	2521.94	61.70	2.4%
6.86_286.1760m/z	286.1760	6.86	172.3	589.17	573.84	587.90	608.36	578.02	589.04	587.72	10.93	1.9%
6.73_282.1084m/z	282.1084	6.73	169.0	389.97	385.22	389.13	397.33	385.23	389.88	389.46	4.05	1.0%
6.70_385.2062m/z	385.2062	6.70	191.8	94.08	92.13	92.41	102.65	84.31	94.18	93.29	5.35	5.7%
6.74_905.4577n	886.4487	6.74	304.4	641.38	647.93	646.12	645.50	616.52	641.74	639.86	10.70	1.7%
6.70_778.3372m/z	778.3372	6.70	260.4	3810.64	3831.27	3739.47	3905.69	3749.77	3810.90	3807.96	55.06	1.4%
6.69_479.2112m/z	479.2112	6.69	207.4	52.98	50.59	55.00	53.41	51.96	52.97	52.82	1.35	2.5%
6.69_839.9280m/z	839.9280	6.69	216.6	116.46	115.76	121.96	109.21	117.55	116.42	116.23	3.75	3.2%
6.69_222.0763m/z	222.0763	6.69	151.4	224.54	222.46	226.72	222.30	226.19	224.42	224.44	1.67	0.7%
6.76_509.2722m/z	509.2722	6.76	217.7	1776.75	1746.56	1774.74	1825.45	1738.74	1776.59	1773.14	27.85	1.6%
6.80_468.2068m/z	468.2068	6.80	202.3	1942.45	1932.38	1918.40	2000.77	1901.19	1942.51	1939.62	30.92	1.6%
6.79_525.2293m/z	525.2293	6.79	213.8	1418.35	1423.99	1401.89	1449.77	1389.53	1418.59	1417.02	18.75	1.3%

6.79_612.3359m/z	612.3359	6.79	241.8	5358.33	5318.04	5256.28	5579.98	5213.95	5358.87	5347.57	116.42	2.2%
6.90_411.1864m/z	411.1864	6.90	196.5	756.97	748.13	755.36	770.77	748.19	756.80	756.04	7.56	1.0%
7.04_545.7732m/z	545.7732	7.04	185.0	381.12	384.62	396.82	339.39	396.72	380.76	379.90	19.30	5.1%
7.04_881.4343m/z	881.4343	7.04	282.5	530.08	523.33	525.00	548.16	517.44	530.09	529.02	9.58	1.8%
7.04_402.6689m/z	402.6689	7.04	155.6	536.26	539.94	526.72	548.94	526.84	536.32	535.84	7.67	1.4%
7.03_291.0977m/z	291.0977	7.03	168.7	416.53	416.15	419.59	406.15	424.98	416.20	416.60	5.61	1.3%
7.08_553.2956m/z	553.2956	7.08	224.2	464.10	459.28	446.36	493.20	448.64	464.17	462.63	15.33	3.3%
7.01_270.1083m/z	270.1083	7.01	161.0	409.02	402.96	416.16	393.43	424.02	408.42	409.00	9.63	2.4%
7.06_368.1681m/z	368.1681	7.06	185.2	378.59	370.49	380.98	379.22	382.25	378.30	378.30	3.76	1.0%
7.06_677.2896m/z	677.2896	7.06	242.7	2411.25	2430.90	2361.34	2481.02	2357.53	2411.81	2408.97	42.03	1.7%
6.94_213.1237m/z	213.1237	6.94	148.6	106.97	101.86	111.74	105.03	107.50	106.88	106.66	2.95	2.8%
6.95_527.2821m/z	527.2821	6.95	222.9	982.96	962.64	962.08	1031.36	958.71	982.87	980.10	24.94	2.5%
6.98_580.2352m/z	580.2352	6.98	220.1	631.54	622.09	637.78	627.09	637.89	631.06	631.24	5.60	0.9%
6.97_357.0888m/z	357.0888	6.97	175.2	1383.48	1390.40	1387.43	1373.06	1380.52	1383.24	1383.02	5.47	0.4%
6.96_802.9614m/z	802.9614	6.96	220.7	325.76	327.81	323.71	325.01	327.56	325.63	325.91	1.42	0.4%
6.95_341.1456m/z	341.1456	6.95	175.6	592.76	579.11	612.93	564.06	612.96	591.95	592.29	17.44	2.9%
4.82_335.6532m/z	335.6532	4.82	160.5	3924.69	3856.05	4012.47	3882.07	3918.98	3922.88	3919.52	48.49	1.2%
4.82_531.3171m/z	531.3171	4.82	230.2	26559.29	26384.82	25531.72	27737.72	26339.96	26550.19	26517.28	647.13	2.4%
4.81_448.2727m/z	448.2727	4.81	219.0	473.25	487.51	440.79	484.73	479.71	473.04	473.17	15.44	3.3%
4.87_449.2742m/z	449.2742	4.87	219.0	44.14	45.45	37.03	51.76	38.02	44.22	43.44	4.91	11.3%
4.73_761.5240m/z	761.5240	4.73	294.0	11772.81	11450.37	12733.44	10493.18	12011.58	11763.44	11704.14	669.95	5.7%
4.77_556.2865m/z	556.2865	4.77	231.6	3806.03	3709.17	3904.27	3738.57	3853.33	3802.68	3802.34	65.51	1.7%
4.83_336.1586n	335.1514	4.83	160.6	17635.87	17175.64	18191.10	17263.96	17777.48	17622.48	17611.09	335.40	1.9%
4.89_706.4660m/z	706.4660	4.89	278.9	742.24	729.88	745.99	743.97	746.45	741.73	741.71	5.57	0.8%
4.95_750.5251m/z	750.5251	4.95	294.1	2275.49	2202.59	2435.68	2110.45	2283.56	2274.06	2263.64	97.84	4.3%
4.30_759.5178m/z	759.5178	4.30	288.0	47585.08	47055.43	49647.86	45075.05	47876.44	47564.03	47467.31	1344.00	2.8%
4.29_773.5340m/z	773.5340	4.29	291.8	145470.77	142061.49	154759.17	135412.67	145946.28	145400.84	144841.87	5728.89	4.0%
4.28_672.4726n	717.4697	4.28	280.7	1707.34	1686.29	1753.01	1615.99	1771.76	1705.02	1706.57	50.04	2.9%
4.36_710.5332n	745.5026	4.36	282.3	587600.03	578220.98	612377.26	562666.78	588869.97	587335.98	586178.50	14793.59	2.5%

4.23_731.4859m/z	731.4859	4.23	276.6	5816.57	5688.83	6037.20	5641.37	5834.45	5813.30	5805.29	126.00	2.2%
4.13_693.4684m/z	693.4684	4.13	273.2	5875.81	5807.47	6255.04	5284.98	5978.02	5872.38	5845.62	289.41	5.0%
4.22_665.3975m/z	665.3975	4.22	262.0	37.06	36.77	34.82	44.00	28.12	37.12	36.32	4.65	12.8%
4.18_733.5025m/z	733.5025	4.18	280.5	113125.98	108623.88	121589.14	104604.69	114672.75	113024.03	112606.74	5248.68	4.7%
4.47_483.2727m/z	483.2727	4.47	225.6	8856.41	8551.69	9187.34	8598.23	9032.98	8847.12	8845.63	223.45	2.5%
4.42_764.5142m/z	764.5142	4.42	289.9	887.39	869.87	884.79	880.82	914.36	886.29	887.26	13.45	1.5%
4.53_509.2886m/z	509.2886	4.53	226.9	44129.07	41996.04	46686.18	42524.15	44596.76	44085.54	44002.96	1516.01	3.4%
4.50_763.5133m/z	763.5133	4.50	286.0	6013.21	5994.30	6281.35	5504.04	6206.01	6007.85	6001.13	247.60	4.1%
5.56_178.0664m/z	178.0664	5.56	129.5	9483.08	9685.63	9064.18	9883.79	9251.20	9487.19	9475.84	267.87	2.8%
5.58_462.2524m/z	462.2524	5.58	216.9	1603.47	1627.37	1547.80	1672.69	1556.44	1604.56	1602.05	42.16	2.6%
5.57_462.2848m/z	462.2848	5.57	202.4	672.37	669.12	680.71	667.23	667.68	672.24	671.56	4.56	0.7%
5.56_199.9942m/z	199.9942	5.56	125.1	212.66	226.16	191.75	225.72	206.03	212.93	212.54	11.79	5.5%
5.46_185.0743m/z	185.0743	5.46	129.1	32681.76	32553.60	33716.80	30528.80	33873.15	32641.18	32665.88	1091.06	3.3%
5.49_357.0793m/z	357.0793	5.49	173.5	6375.12	6526.98	6036.04	6757.69	6094.25	6380.87	6361.82	245.72	3.9%
5.51_530.2778m/z	530.2778	5.51	213.7	138.00	144.38	139.38	130.55	135.25	138.20	137.63	4.18	3.0%
5.49_530.2771m/z	530.2771	5.49	213.7	111.69	114.36	113.13	109.76	103.98	111.83	110.79	3.36	3.0%
5.77_770.5475m/z	770.5475	5.77	291.8	33.78	29.83	38.42	29.53	34.51	33.71	33.30	3.01	9.0%
5.76_466.3078m/z	466.3078	5.76	204.1	7045.50	6883.93	7184.02	7014.37	7045.65	7041.65	7035.85	87.29	1.2%
5.74_592.2749m/z	592.2749	5.74	227.2	413.81	419.53	416.85	411.74	405.00	414.05	413.50	4.53	1.1%
5.73_464.4977m/z	464.4977	5.73	204.2	3232.71	3153.62	3318.54	3191.22	3241.33	3230.76	3228.03	50.48	1.6%
5.72_464.9111m/z	464.9111	5.72	202.4	335.83	324.58	359.47	311.22	339.18	335.58	334.31	14.66	4.4%
5.72_464.6266m/z	464.6266	5.72	204.2	575.12	562.43	579.24	577.71	578.55	574.68	574.62	5.70	1.0%
5.69_359.7356m/z	359.7356	5.69	153.3	1839.92	1761.63	1948.96	1750.61	1868.68	1838.14	1834.66	66.65	3.6%
5.63_691.3412m/z	691.3412	5.63	248.2	5081.09	4955.93	5201.39	5025.70	5106.08	5077.54	5074.62	74.83	1.5%
5.67_467.1597m/z	467.1597	5.67	216.8	1372.53	1439.57	1263.49	1460.92	1311.60	1374.41	1370.42	68.18	5.0%
5.20_248.0792m/z	248.0792	5.20	140.5	22338.00	20280.95	24770.95	20368.46	23014.54	22300.02	22178.82	1545.80	7.0%
5.21_691.4779n	690.4703	5.21	275.2	2132.02	2124.96	2109.50	2134.74	2162.17	2130.38	2132.29	15.68	0.7%
5.19_248.5806m/z	248.5806	5.19	140.5	4384.25	3875.57	4977.52	3860.71	4551.13	4375.65	4337.47	387.22	8.9%
5.18_514.2843m/z	514.2843	5.18	210.4	1368561.17	1236501.17	1536540.94	1199031.93	1432121.09	1365378.35	1356355.78	113690.64	8.4%

5.10_702.4710m/z	702.4710	5.10	277.0	6129.19	6261.29	5939.08	6209.89	6115.83	6129.35	6130.77	100.22	1.6%
5.14_652.3928m/z	652.3928	5.14	268.0	1823.03	1766.85	1856.54	1831.50	1819.81	1821.98	1819.95	26.79	1.5%
5.12_255.0860m/z	255.0860	5.12	141.8	1224.74	1106.66	1367.13	1108.76	1267.20	1222.72	1216.20	90.41	7.4%
5.46_371.1565m/z	371.1565	5.46	190.4	10991.34	10921.79	11485.84	10048.12	11414.28	10977.22	10973.10	468.35	4.3%
5.78_468.2730m/z	468.2730	5.78	222.2	5114.27	5058.88	5170.76	5068.69	5144.71	5111.42	5111.46	39.21	0.8%
5.30_498.2905m/z	498.2905	5.30	208.9	27.80	26.45	30.16	26.04	27.65	0.00	23.02	10.38	45.1%
