# Supplementary material for: Choosing an Optimal Sample Preparation in Caulobacter crescentus for Untargeted Metabolomics Approaches
Source: Metabolites. 2019 Sep 20;9(10):193. doi: 10.3390/metabo9100193 (PMC6836107; doi:10.3390/metabo9100193)
Supplement: Supplementary file 1 [file metabolites-09-00193-s001.zip › Supp Data/Table S2.pdf]

Name	Adapted Name	Observed Rt (min)	Observed m/z	Observed CCS	Observed adduct(s)	CAS Identifier	Confirmation Rt	Confirmation MS/MS	Confirmation CCS	MS/MS & CCS	Chemical Class
L-Glutamine	L-Glutamine	9.19	145.0615	130.3	M-H	56-85-9	v	N/A	v		Amino acids/derivatives
Inosine 5'-Phosphate	IMP	9.19	347.0403	170.3	M-H	131-99-7	v	v	v	v	Nucleotides/analogs
Citric Acid	Citric Acid	10.07	191.0195	125.6	M-H	77-92-9	v	N/A	v		Organic acids
N-Acetylneuraminic Acid	N-AN Acid	8.37	290.0877	158.6	M-H2O-H	131-48-6	v	v	v	v	Organic acids
Uric Acid	Uric Acid	8.45	167.0210	123.9	M-H	69-93-2	v	N/A	v		Organic acids
Cytidine	Cytidine	8.16	242.0778	153.8	M-H	65-46-3	v	v	v	v	Nucleosides/analogs
Inosine	Inosine	7.01	267.0731	156.1	M-H	58-63-9	v	v	v	v	Nucleosides/analogs
L-Isoleucine	L-Isoleucine	7.3	130.0872	130.1	M-H	73-32-5	v	N/A	v		Amino acids/derivatives
L-Glutamic Acid	Glutamic Acid	8.98	147.0527	123.9	M-H2O-H, M-H	56-86-0	v	v	v	v	Amino acids/derivatives
N-Acetyl-D-Glucosamine	N-AG	8.64	202.0717	142.6	M-H2O-H	7512-17-6	v	N/A	v		Carbohydrates
Gluconic Acid	Gluconic Acid	8.92	195.0506	131.7	M-H	526-95-4	v	v	v	v	Organic acids
Quinic Acid	Quinic Acid	8.71	237.0613	145.8	M+FA-H	77-95-2	v	N/A	v		Organic acids
Adenine	Adenine	5.9	134.0472	118.6	M-H	6055-72-7	v	v	v	v	Nucleosides/analogs
Xanthine	Xanthine	6.67	151.0262	120.3	M-H	69-89-6	v	v	v	v	Nucleosides/analogs
L-Tryptophan	L-Tryptophan	7.6	203.0825	149.1	M-H	73-22-3	v	v	v	v	Amino acids/derivatives
Uridine 5'-Monophosphate	U-5MP	8.97	323.0285	160.9	M-H	58-97-9	v	v	v	v	Nucleotides/analogs
Uridine	Uridine	6.33	243.0622	148.8	M-H	58-96-8	v	v	v	v	Nucleosides/analogs
Fructose 1,5-Biphosphate	F1,5-BP	11.08	338.9880	152.2	M-H	488-69-7	v	N/A	v		Phosphates
Carnosine	Carnosine	9.79	225.0987	149.6	M-H	305-84-0	v	N/A	v		Amino acids/derivatives
Shikimic Acid	Shikimic Acid	8.68	219.0508	141.7	M+FA-H	138-59-0	v	N/A	v		Organic acids
L-Phenylalanine	Phenylalanine	7.09	164.0717	138.5	M-H	63-91-2	v	v	v	v	Amino acids/derivatives
Hypoxanthine	Hypoxanthine	6.38	135.0313	118.5	M-H	68-94-0	v	v	v	v	Nucleosides/analogs
Guanosine	Guanosine	7.84	282.0840	158.9	M-H	118-00-3	v	v	v	v	Nucleosides/analogs
L-Tyrosine	Tyrosine	8.27	180.0665	142.3	M-H	60-18-4	v	v	v	v	Amino acids/derivatives
L-Asparagine	L-Asparagine	9.34	131.0459	197.9	M-H	70-47-3	v	v	N/A		Amino acids/derivatives
Guanine	Guanine	7.49	150.0415	123.5	M-H	73-40-5	v	N/A	v		Nucleosides/analogs
DAMP	DAMP	8.25	330.0604	170.8	M-H	653-63-4	v	v	v	v	Nucleotides/analogs
(R,R)-L-(+)-Tartaric Acid	(R,R)-T Acid	10.12	149.0088	118.9	M-H	87-69-4	v	N/A	v		Organic acids

N-Acetyl-DL-Tryptophan	N-A-Tryptophan	5.88	245.0930	157.0	M-H	87-32-1	v	v	v	v	Organic acids
D-Glucose 6-Phosphate	Glucose 6-P	9.61	259.0217	144.9	M-H	56-73-5	v	N/A	v		Phosphates
LL-2,6-Diaminoheptanedioic Acid	2,6-DH Acid	10.62	189.0880	136.9	M-H	583-93-7	v	N/A	v		Amino acids/derivatives
Alpha-Aminosdipic Acid	Aminoadipate	9.08	160.0614	129.1	M-H	542-32-5	v	v	v	v	Amino acids/derivatives
D-Glucosamine 6-Phosphate	G6P	9.39	214.0480	142.0	M-CO2-H	70442-23-8	v	v	v	v	Phosphates
D-(+)-Trehalose	Trehalose	9.34	377.0850	172.9	M+Cl	6138-23-4	v	v	v	v	Carbohydrates
Adenosine 5'-Diphosphate	ADP	10.28	426.0207	183.8	M-H	20398-34-9	v	v	v	v	Nucleotides/analogs
2-Deoxy-D-Glucose	2-Deoxy-Glucose	8.2	163.0606	128.9	M-H	154-17-6	v	N/A	v		Carbohydrates
4-Pyridoxic Acid	4-Pyridoxic Acid	2.16	164.0347	132.0	M-H2O-H	82-82-6	v	N/A	v		Organic acids
3-Hydroxy-3-Methylglutaric Acid	3-H-3-Methylglutarate	7.8	161.0450	127.5	M-H	503-49-1	v	N/A	v		Organic acids
Xanthosine	Xanthosine	8.24	283.0673	158.9	M-H	146-80-5	v	N/A	v		Nucleosides/analogs
D-Galactaric Acid	Galactaric Acid	8.48	165.0395	125.6	M-CO2-H	526-99-8	v	N/A	v		Organic acids
L-Histidine	L-Histidine	9.13	154.0622	128.0	M-H	71-00-1	v	v	v	v	Amino acids/derivatives
Guanosine 5'-Triphosphate	GTP	11.78	521.9850	195.9	M-H	36051-31-7	v	v	v	v	Nucleotides/analogs
2'-Deoxyguanosine 5'-Triphosphate	2'-DG-5'TP	10.91	505.9879	189.2	M-H	93919-41-6	v	v	v	v	Nucleotides/analogs
Inosine 5'-Diphosphate	IDP	9.47	427.0068	176.9	M-H	81012-88-6	v	v	v	v	Nucleotides/analogs
Adenosine 5'-Monophosphate	AMP	8.56	347.0631n	172.1	M-H, 2M-H	4578-31-8	v	v	v	v	Nucleotides/analogs
Uridine 5'-Diphosphoglucose	UDP-Glucose	9.78	565.0474	213.1	M-H	28053-08-9	v	v	v	v	Nucleotides/analogs
2'-Deoxyguanosine 5'-Diphosphate	dGDP	10.57	408.0130	172.2	M-H2O-H	102783-74-4	v	N/A	v		Nucleotides/analogs
Cytidine 2',3'-Cyclic Monophosphate	cCMP	7.28	260.0440	153.1	M-CO2-H	15718-51-1	v	v	v	v	Nucleotides/analogs
D-(-)-3-Phosphoglyceric Acid	3-PG Acid	11.12	220.9626	143.3	M+Cl	80731-10-8	v	N/A	v		Phosphates
Thymidine 5'-Monophosphate	dTMP	8.21	321.0492	162.6	M-H	365-07-1	v	v	v	v	Nucleotides/analogs
N-Alpha-Acetyl-L-Lysine	A-L-Lysine	9.03	187.1084	145.1	M-H	1946-82-3	v	v	v	v	Amino acids/derivatives
Adenosine 5'-Triphosphate	ATP	10.27	505.9880	189.2	M-H	34369-07-8	v	v	v	v	Nucleotides/analogs
2'-Deoxyguanosine	2'-DG	7.01	266.0887	159.5	M-H	961-07-9	v	v	v	v	Nucleosides/analogs
5-Aminoimidazole-4-Carboxamide-1-Beta-D-Ribofuranosyl 5'-Monophosphate	AICAR	8.58	383.0600	176.2	M+FA-H	3031-94-5	v	v	v	v	Nucleotides/analogs
Uridine 5'-Diphospho-N-Acetylgalactosamine	U-5'DP-AG	9.44	606.0738	225.2	M-H	108320-87-2	v	v	v	v	Nucleotides/analogs
Guanosine 3',5'-Cyclic Monophosphate	cGMP	8.47	344.0398	170.4	M-H	40732-48-7	v	v	v	v	Nucleotides/analogs
S-(5'-Adenosyl)-L-Homocysteine	S-5'-Homocysteine	8.65	383.1135	186.6	M-H	979-92-0	v	v	v	v	Not defined

Guanosine 5'-Diphosphate	GDP	10.71	442.0167	181.8	M-H	43139-22-6	v	v	v	v	Nucleosides/analogs
Uridine 5'-Diphosphoglucuronic Acid	UDP Glucuronic Acid	11.17	579.0267	212.8	M-H	63700-19-6	v	v	v	v	Nucleotides/analogs
Cytidine 5'-Diphosphate	CDP	10.38	402.0097	172.3	M-H	34393-59-4	v	v	v	v	Nucleotides/analogs
Guanosine 5'-Monophosphate	GMP	9.43	362.0497	169.9	M-H	85-32-5	v	v	v	v	Nucleotides/analogs
Methyl-Beta-D-Galactoside	M-B-Galactoside	6.88	193.0727	139.9	M-H	1824-94-8	v	v	v	v	Carbohydrates
Cytidine 5'-Monophosphate	5'-CMP	9.34	322.0443	162.6	M-H	63-37-6	v	v	v	v	Nucleotides/analogs
Glutathione	GSH reduced	8.84	307.0826n	161.4	M-H2O-H, M-H	70-18-8	v	v	v	v	Amino acids/derivatives
N-Acetyl-D-Galactosamine	N-A-Galactosamine	8.81	202.0714	139.4	M-H2O-H	1811-31-0	v	v	v	v	Carbohydrates
N-Acetyl-DL-Glutamic Acid	N-A-Glutamic Acid	9.02	188.0563	136.9	M-H	1188-37-0	v	v	v	v	Organic acids
D-Pantothenic Acid	Pantothenic Acid	6.12	218.1031	146.7	M-H	137-08-6	v	v	v	v	Organic acids
Adenosine	Adenosine	5.99	312.0946	164.6	M+FA-H	58-61-7	v	v	v	v	Nucleosides/analogs
D-Lactose	Lactose	10.14	341.1074	170.5	M-H	64044-51-5	v	v	v	v	Carbohydrates
Cytidine 5'-Triphosphate	CTP	11.22	481.9749	182.7	M-H	36051-68-0	v	v	v	v	Phosphates
Cis-4-Hydroxy-Proline	4-H-Proline	8.67	176.0562	125.3	M-H	2584-71-6	v	N/A	v		Amino acids/derivatives
Adenosine 5'-Diphosphoribose	ADP-Ribose	8.85	540.0537	213.5	M-H2O-H	68414-18-6	v	v	v	v	Nucleotides/analogs
Ophthalmic Acid	Ophthalmic Acid	8.58	288.1197	160.4	M-H	495-27-2	v	v	v	v	Amino acids/derivatives
Coenzyme A	CoA	8.94	766.1078	249.0	M-H	55672-92-9	v	v	v	v	Phosphates
CDP-Ethanolamine	CDP-Ethanolamine	9.13	445.0526	178.2	M-H	3036-18-8	v	v	v	v	Nucleotides/analogs
Stachyose	Stachyose	9.46	711.2175	247.9	M+FA-H	54261-98-2	v	v	v	v	Carbohydrates
D-Ribose-5-Phosphate	Ribose 5-P	9.29	211.0002	134.1	M-H2O-H	18265-46-8	v	N/A	v		Phosphates
N-Acetyl-DL-Serine	N-A-L-Serine	7.65	128.0346	178.3	M-H2O-H	97-14-3	v	N/A	v		Organic acids
Glycerol 2-Phosphate	G2-P	9.03	171.0061	125.2	M-H	819-83-0	v	N/A	v		Phosphates
N-Acetylproline	N-Acetylproline	5.75	156.0661	134.3	M-H	68-95-1	v	N/A	v		Amino acids/derivatives
Glycocholic Acid	Gcholic Acid	5.72	465.3091n	204.2	M-H, 2M-H	475-31-0	v	v	v	v	Organic acids
Glycochenodeoxycholic Acid	Gdeoxycholic Acid	5.14	448.3063	200.9	M-H	640-79-9	v	v	v	v	Steroids
Methyl-Vanillic Acid	M-Vanillate	2.17	163.0407	135.3	M-H2O-H	3943-74-6	v	N/A	v		Organic acids
4-Hydroxybenzaldehyde	4-Hydroxybenzaldehyde	1.56	121.0293	118.3	M-H	123-08-0	v	N/A	v		Not defined
Pterin	Pterin	6.71	162.0418	125.8	M-H	2236-60-4	v	N/A	v		Nucleosides/analogs
Azelaic Acid	Azelaic Acid	4.15	187.0972	140.2	M-H	123-99-9	v	v	v	v	Lipid/derivatives

Suberic Acid	Suberic Acid	5.79	173.0816	134.6	M-H	505-48-6	v	N/A	v		Lipid/derivatives
Sebaic Acid	Sebaic Acid	2.4	201.1139	145.9	M-H	111-20-6	v	N/A	v		Organic acids
D-(+)-Xylose	Xylose	8.25	299.0998	161.7	2M-H	58-86-6	v	N/A	v		Carbohydrates
Sucrose	Sucrose	8.9	387.1141	174.4	M+FA-H	57-50-1	v	v	v	v	Carbohydrates
Alpha-D-Glucose	Glucose	8.57	180.0630n	131.0	M-H2O-H, M-H	492-62-6	v	v	N/A		Carbohydrates
Palatinose	Palatinose	8.9	342.1161n	168.8	M-H, M+Cl	343336-76-5	v	v	v	v	Carbohydrates
Petroselinic Acid	P Acid	1.67	281.2484	179.4	M-H	593-39-5	v	N/A	v		Lipid/derivatives
Chenodeoxycholic Acid	Cdcholic Acid	2.62	391.2872	207.7	M-H	474-25-9	v	v	v	v	Steroids
Myristic Acid	Myristic Acid	1.13	227.2020	166.3	M-H	544-63-8	v	N/A	v		Lipid/derivatives
Rosmarinic Acid	Rosmarinic Acid	7.98	395.0540	177.6	M+Cl	20283-92-5	v	N/A	v		Organic acids
Taurolitocholic Acid	Tlcholic Acid	1.56	482.2943	209.2	M-H	6042-32-6	v	v	v	v	Steroids
Hexadecanoic Acid	Hexadecanoic Acid	1.28	253.2164	170.2	M-H	373-49-9	v	N/A	v		Lipid/derivatives
Palmitic Acid	Palmitic Acid	1.66	255.2323	173.6	M-H	57-10-3	v	N/A	v		Lipid/derivatives
Eicosapentaenoic Acid	EPA	0.96	347.2240	189.4	M+FA-H	10417-94-4	v	N/A	v		Organic acids
Cholic Acid	Cholic Acid	2.28	407.2797	205.5	M-H	81-25-4	v	v	v	v	Steroids
Oleic Acid	Oleic Acid	1.31	281.2483	177.7	M-H	112-80-1	v	N/A	v		Lipid/derivatives
Stearic Acid	Stearic Acid	0.73	283.2640	181.1	M-H	57-11-4	v	N/A	v		Lipid/derivatives
Cortisone	Cortisone	1.93	405.1906	202.0	M+FA-H	53-06-5	v	v	v	v	Steroids
Acetyl-CoA	Acetyl-CoA	8.48	808.1185	254.2	M-H	72-89-9	v	v	v	v	Phosphates
Succinyl-CoA	Succinyl-CoA	9.91	866.1197	253.5	M-H	604-98-8	v	v	v	v	Phosphates
