# Supplementary material for: Choosing an Optimal Sample Preparation in Caulobacter crescentus for Untargeted Metabolomics Approaches
Source: Metabolites. 2019 Sep 20;9(10):193. doi: 10.3390/metabo9100193 (PMC6836107; doi:10.3390/metabo9100193)
Supplement: Supplementary file 1 [file metabolites-09-00193-s001.zip › Supp Data/Table S3.pdf]

Compound	Adapted Name	Cells retrieval VIP^2	Solvent VIP^2	Cells disruption VIP^2	Residuals VIP^2
7.80_161.0450m/z	3-H-3-Methylglutarate	3.46868525	0.01176429	0.09224117	0.01341271
8.20_163.0606m/z	2-Deoxy-Glucose	3.46362326	0.00000289	0.05417190	0.00003147
8.57_180.0630m/z	Glucose	3.42566462	0.01145761	0.24651233	0.11389171
7.65_128.0346m/z	N-A-L-Serine	3.25602784	0.17235607	0.13661398	0.00183962
1.56_482.2943m/z	Tlcholic Acid	3.01713783	0.04734984	0.57872987	0.13098568
8.25_299.0998m/z	Xylose	2.83096290	0.15783802	0.18433974	0.14535641
8.16_242.0778m/z	Cytidine	2.80868490	0.32064218	0.29147040	0.20864985
5.72_465.3091m/z	Gcholic Acid	2.71964561	0.00545484	0.29419280	1.03150733
8.27_180.0665m/z	Tyrosine	2.53521879	0.00667159	0.25269603	1.37069629
6.88_193.0727m/z	M-B-Galactoside	2.50329388	0.71181877	0.03345336	1.19994891
8.48_165.0395m/z	Galactaric Acid	2.46687955	0.00813805	0.14675500	0.00000408
8.25_330.0604m/z	DAMP	2.46546475	0.59181513	0.00032268	0.86058630
8.21_321.0492m/z	dTMP	2.46059535	0.57820935	0.00038940	1.30332678
8.45_167.0210m/z	Uric Acid	2.44693330	0.45644525	0.18421922	1.39783043
7.49_150.0415m/z	Guanine	2.44260884	0.02225635	0.47507204	1.23608460
5.14_448.3063m/z	Gdeoxycholic Acid	2.41421550	0.02490126	0.55638494	0.99961043
8.56_347.0631m/z	AMP	2.19595820	1.25405292	0.29173418	0.68912301
9.08_160.0614m/z	Aminoadipate	2.11489499	1.02450991	0.13053063	0.01460610
1.28_253.2164m/z	Hexadecanoic Acid	1.93067409	0.26606166	1.04710530	1.84865760
8.67_176.0562m/z	4-H-Proline	1.91664124	0.06908115	1.30458967	0.59375977
2.28_407.2797m/z	Cholic Acid	1.89582348	0.09548776	0.03126367	1.07473079
1.31_281.2483m/z	Oleic Acid	1.86782879	0.18228491	1.70044419	0.27663385
6.67_151.0262m/z	Xanthine	1.86772626	0.01122609	0.10819995	2.20205381
8.58_383.0600m/z	AICAR	1.86144855	0.01375718	2.04269628	0.01106107
7.30_130.0872m/z	L-Isoleucine	1.74438855	0.38583656	0.09664831	1.95162715
8.37_290.0877m/z	N-AN Acid	1.69476027	0.15218123	0.25975874	1.24991897
7.01_266.0887m/z	2'-DG	1.65546365	0.00193781	0.52264833	1.88643172
6.33_243.0622m/z	Uridine	1.57539887	0.09628356	0.03456668	2.63257980

8.24_283.0673m/z	Xanthosine	1.55638633	1.28836640	0.26857230	0.45437956
5.99_312.0946m/z	Adenosine	1.52791743	0.00017638	0.02309745	1.99851292
8.47_344.0398m/z	cGMP	1.52566588	0.00376243	0.04461547	1.06356761
7.01_267.0731m/z	Inosine	1.46429324	0.03546149	0.00281861	1.36682999
6.71_162.0418m/z	Pterin	1.40056870	1.37977873	0.38455643	0.29895745
8.94_766.1078m/z	CoA	1.39970051	0.34976369	0.40563862	0.54267514
8.64_202.0717m/z	N-AG	1.36155201	2.34356122	0.18980384	0.03607112
11.78_521.9850m/z	GTP	1.34494907	1.55392667	2.00298331	0.06153514
10.12_149.0088m/z	(R,R)-T Acid	1.31242820	0.83186698	1.51987516	1.80364212
1.56_121.0293m/z	4-Hydroxybenzaldehyde	1.31127242	0.05362682	0.26962370	0.25143546
8.97_323.0285m/z	U-5MP	1.20249457	0.01435281	0.20276636	2.21808569
8.92_195.0506m/z	Gluconic Acid	1.19864438	0.00509960	2.68140948	0.55930942
10.62_189.0880m/z	2,6-DH Acid	1.14522221	0.66518393	0.26745513	0.12682773
9.61_259.0217m/z	Glucose 6-P	1.09913520	0.04815972	1.55761794	1.67947604
8.84_307.0826m/z	GSH reduced	1.08114255	2.55136407	0.04165907	0.85239826
6.12_218.1031m/z	Pantothenic Acid	1.03615986	0.41092397	0.49412599	3.01934535
8.48_808.1185m/z	Acetyl-CoA	1.01481412	0.15368956	3.83806388	0.80734075
7.28_260.0440m/z	cCMP	0.95037776	3.46961957	0.04688985	0.03673490
8.65_383.1135m/z	S-5'-Homocysteine	0.94721424	3.71277575	1.48744034	0.52800490
9.79_225.0987m/z	Carnosine	0.92066342	1.48262629	0.10475652	0.04854229
2.62_391.2872m/z	Cdcholic Acid	0.86726224	0.03445489	0.00168464	1.24080678
1.13_227.2020m/z	Myristic Acid	0.78317685	0.72516786	0.36080622	0.02030236
7.84_282.0840m/z	Guanosine	0.77275588	0.11252423	0.19619517	2.32058016
9.03_171.0061m/z	G2-P	0.75585608	0.06397708	0.28711671	2.36860699
9.91_866.1197m/z	Succinyl-CoA	0.73811359	0.69062011	0.42239247	1.80744609
9.19_347.0403m/z	IMP	0.68568865	2.12657899	0.73259156	0.86776066
11.17_579.0267m/z	UDP Glucuronic Acid	0.62581794	0.20432928	0.77952590	1.05994338
10.07_191.0195m/z	Citric Acid	0.60839283	0.49059781	0.66726329	1.70654989
5.75_156.0661m/z	N-Acetylproline	0.60804443	0.00607687	0.02672831	1.68937034

9.78_565.0474m/z	UDP-Glucose	0.60086705	0.42658884	0.64990332	1.60314255
11.22_481.9749m/z	CTP	0.56323980	0.24960903	4.27117230	0.38404867
10.27_505.9880m/z	ATP	0.55997788	1.13929533	1.71168917	1.43230092
8.98_147.0527m/z	Glutamic Acid	0.55136939	1.03146863	1.17621460	3.07249210
8.71_237.0613m/z	Quinic Acid	0.53997222	0.26793241	0.92470503	0.30609615
5.90_134.0472m/z	Adenine	0.50931024	0.10624432	0.73363623	2.59431057
9.39_214.0480m/z	G6P	0.48911847	4.44988783	0.00070296	0.07784260
10.57_408.0130m/z	dGTP	0.48250751	0.02545136	0.28237185	1.77076627
2.16_164.0347m/z	4-Pyridoxic Acid	0.45847901	0.35040466	0.48099455	0.01613514
5.79_173.0816m/z	Suberic Acid	0.44567289	0.00349885	1.07550061	2.02263481
8.81_202.0714m/z	N-A-Galactosamine	0.43629264	0.56918837	1.32261559	0.02068953
9.34_131.0459m/z	L-Asparagine	0.41567668	5.99473774	0.81606554	0.07340127
9.34_322.0443m/z	5'-CMP	0.37033478	5.68299272	1.05231686	0.87920734
10.14_341.1074m/z	Lactose	0.36536044	0.08378960	0.01229174	2.28456529
8.85_540.0537m/z	ADP-Ribose	0.34547079	0.43052083	0.93849662	1.83153693
10.91_505.9879m/z	2'-DG-5'TP	0.33855849	1.72853231	3.14498056	0.84577157
0.96_347.2240m/z	EPA	0.32930590	0.26226562	0.80286390	1.12762131
2.17_163.0407m/z	M-Vanillate	0.31751285	0.76827743	0.79768756	1.17651517
7.98_395.0540m/z	Rosmarinic Acid	0.31610264	1.04061524	0.26132588	0.51706858
7.60_203.0825m/z	L-Tryptophan	0.30064645	0.11849467	0.00114710	5.47767301
9.03_187.1084m/z	A-L-Lysine	0.27787065	0.81010154	0.10820914	0.97556693
8.68_219.0508m/z	Shikimic Acid	0.25926975	0.45723802	1.14063557	0.06650736
9.43_362.0497m/z	GMP	0.25402276	3.19502869	0.84911279	0.66778232
8.58_288.1197m/z	Ophthalmic Acid	0.25036399	2.82368104	2.12855879	1.79105130
9.02_188.0563m/z	N-A-Glutamic Acid	0.23231893	0.04864106	4.11798211	1.55862237
2.40_201.1139m/z	Sebaic Acid	0.22414969	0.31603789	0.88807270	0.36405429
9.44_606.0738m/z	U-5'DP-AG	0.17121599	2.28407865	0.71034699	1.29952778
9.13_445.0526m/z	CDP-Ethanolamine	0.16252590	0.54367600	0.64392157	0.44224714
1.93_405.1906m/z	Cortisone	0.15728966	0.51780021	0.62317575	0.17622907

9.34_377.0850m/z	Trehalose	0.07921810	4.09660880	2.35142072	0.10790298
10.28_426.0207m/z	ADP	0.06811882	0.61377224	5.06489550	0.25649931
5.88_245.0930m/z	N-A-Tryptophan	0.05676709	0.21322149	0.47871174	1.21234963
9.19_145.0615m/z	L-Glutamine	0.04432390	6.19707062	0.05472836	0.77510155
11.08_338.9880m/z	F1,5-BP	0.03576518	2.34834523	1.71993763	1.26993471
7.09_164.0717m/z	Phenylalanine	0.03442421	0.65623605	0.44889659	5.61129843
8.90_342.1161m/z	Palatinose	0.03190293	0.12444702	1.25362785	1.46749422
10.38_402.0097m/z	CDP	0.03091930	0.76470466	2.77774900	0.57477245
8.90_387.1141m/z	Sucrose	0.02974136	0.12652975	1.18727142	1.61021322
6.38_135.0313m/z	Hypoxanthine	0.02739844	0.00021488	0.61266352	5.66298107
10.71_442.0167m/z	GDP	0.02482506	0.11032960	4.01288886	0.56563686
11.12_220.9626m/z	3-PG Acid	0.02170693	1.04293926	0.00474432	0.00021668
0.73_283.2640m/z	Stearic Acid	0.01173910	0.11067049	6.04793135	0.00000002
9.47_427.0068m/z	IDP	0.00450126	2.95296687	1.37812381	0.02745668
9.29_211.0002m/z	Ribose 5-P	0.00317117	5.70745543	1.09872813	0.05531789
1.67_281.2484m/z	P Acid	0.00274160	0.60567748	8.25198594	0.01104074
4.15_187.0972m/z	Azelaic Acid	0.00112724	0.03832652	1.12499989	0.53019414
1.66_255.2323m/z	Palmitic Acid	0.00056337	0.38543503	8.21721514	0.01199068
9.46_711.2175m/z	Stachyose	0.00023972	1.16867480	2.75061704	0.34787345
9.13_154.0622m/z	L-Histidine	0.00000299	9.70262545	0.17003288	0.13765816
