# Supplementary material for: Choosing an Optimal Sample Preparation in Caulobacter crescentus for Untargeted Metabolomics Approaches
Source: Metabolites. 2019 Sep 20;9(10):193. doi: 10.3390/metabo9100193 (PMC6836107; doi:10.3390/metabo9100193)
Supplement: Supplementary file 1 [file metabolites-09-00193-s001.zip › Supp Data/Table S4.pdf]

Sample	Cells retrieval	Solvent	Cells disruption	tp1	tp2	tp3	to
Centri CHCl3 Beads 1	Centri	CHCl3	Beads	0.1882	0.1680	0.1110	0.3427
Centri CHCl3 Beads 2	Centri	CHCl3	Beads	0.1376	0.1187	0.1098	-0.2191
Centri CHCl3 Beads 3	Centri	CHCl3	Beads	0.1891	0.1025	0.1443	0.1305
Centri MeOH Beads 1	Centri	MeOH	Beads	0.1571	-0.1258	0.1246	0.3493
Centri MeOH Beads 2	Centri	MeOH	Beads	0.1316	-0.0673	0.0849	-0.2208
Centri MeOH Beads 3	Centri	MeOH	Beads	0.1554	-0.1044	0.1322	-0.1348
Filter CHCl3 Beads 1	Filter	CHCl3	Beads	-0.1627	0.1255	0.1250	0.2298
Filter CHCl3 Beads 2	Filter	CHCl3	Beads	-0.1553	0.0803	0.0654	-0.0234
Filter CHCl3 Beads 3	Filter	CHCl3	Beads	-0.1291	0.0326	0.1533	-0.1489
Filter MeOH Beads 1	Filter	MeOH	Beads	-0.1118	-0.0410	0.0864	-0.2506
Filter MeOH Beads 2	Filter	MeOH	Beads	-0.2196	-0.1346	0.1119	0.0471
Filter MeOH Beads 3	Filter	MeOH	Beads	-0.1805	-0.1544	0.1917	-0.1019
Centri CHCl3 F_T 1	Centri	CHCl3	F/T	0.1788	0.1435	-0.1053	-0.3074
Centri CHCl3 F_T 2	Centri	CHCl3	F/T	0.1770	0.1481	-0.1292	-0.3619
Centri CHCl3 F_T 3	Centri	CHCl3	F/T	0.1863	0.1276	-0.1222	0.0423
Centri MeOH F_T 1	Centri	MeOH	F/T	0.2445	-0.1732	-0.1405	0.1868
Centri MeOH F_T 2	Centri	MeOH	F/T	0.2659	-0.2444	-0.1330	0.1938
Centri MeOH F_T 3	Centri	MeOH	F/T	0.1917	-0.0932	-0.0766	-0.0015
Filter CHCl3 F_T 1	Filter	CHCl3	F/T	-0.2301	0.2753	-0.1486	0.3471
Filter CHCl3 F_T 2	Filter	CHCl3	F/T	-0.1805	0.1325	-0.1223	-0.0662
Filter CHCl3 F_T 3	Filter	CHCl3	F/T	-0.1993	0.1201	-0.0812	0.0343
Filter MeOH F_T 1	Filter	MeOH	F/T	-0.1822	-0.1463	-0.1706	-0.1911
Filter MeOH F_T 2	Filter	MeOH	F/T	-0.2530	-0.1514	-0.1342	0.1238
Filter MeOH F_T 3	Filter	MeOH	F/T	-0.1991	-0.1386	-0.0769	-0.0001
