# Supplementary material for: Choosing an Optimal Sample Preparation in Caulobacter crescentus for Untargeted Metabolomics Approaches
Source: Metabolites. 2019 Sep 20;9(10):193. doi: 10.3390/metabo9100193 (PMC6836107; doi:10.3390/metabo9100193)
Supplement: Supplementary file 1 [file metabolites-09-00193-s001.zip › Supp Data/Table S5.pdf]

Compound	Adapted Name	pp1	pp2	pp3	po
8.20_163.0606m/z	2-Deoxy-Glucose	-5.0965	-0.0392	-0.2964	0.0088
8.57_180.0630m/z	Glucose	-5.0706	-0.1892	-0.9428	0.5311
7.80_161.0450m/z	3-H-3-Methylglutarate	-5.0675	0.1847	-0.5706	0.1822
1.56_482.2943m/z	Tlcholic Acid	-4.9201	0.0408	-1.7274	0.5695
7.65_128.0346m/z	N-A-L-Serine	-4.8777	-0.8124	-0.6343	-0.0675
5.72_465.3091m/z	Gcholic Acid	-4.7150	0.0540	-0.9062	1.5982
8.25_299.0998m/z	Xylose	-4.5881	-1.3152	-0.9023	0.6000
5.14_448.3063m/z	Gdeoxycholic Acid	-4.5629	0.2266	-1.3953	1.5733
8.16_242.0778m/z	Cytidine	-4.5525	-1.3262	-1.3837	0.7188
6.88_193.0727m/z	M-B-Galactoside	-4.4710	-1.4135	-0.5094	1.7238
7.49_150.0415m/z	Guanine	-4.4023	0.7359	1.7152	1.7495
8.27_180.0665m/z	Tyrosine	-4.3718	0.2472	-0.7744	1.8423
8.48_165.0395m/z	Galactaric Acid	-4.1435	-0.9627	-0.7489	0.0032
9.08_160.0614m/z	Aminoadipate	-4.0661	-1.9071	-1.2326	-0.1902
8.58_383.0600m/z	AICAR	-3.8789	-0.4221	-3.0328	0.1655
8.67_176.0562m/z	4-H-Proline	-3.8702	-0.8673	-2.5240	1.2126
2.28_407.2797m/z	Cholic Acid	-3.8474	0.8321	-0.2019	1.6314
6.67_151.0262m/z	Xanthine	-3.7928	-0.1146	-1.0626	2.3351
8.24_283.0673m/z	Xanthosine	-3.5638	-2.7524	-1.0375	1.0607
8.37_290.0877m/z	N-AN Acid	-3.4722	-1.6112	-0.7389	1.7593
7.01_267.0731m/z	Inosine	-3.4282	-0.6847	1.1078	1.8397
1.56_121.0293m/z	4-Hydroxybenzaldehyde	-3.4105	0.3388	-0.7422	0.7891
10.12_149.0088m/z	(R,R)-T Acid	-3.3529	2.5490	-2.6891	2.1134
8.47_344.0398m/z	cGMP	-3.3455	-0.8991	1.5142	1.6229
11.78_521.9850m/z	GTP	-3.1535	2.2483	-3.1684	0.3904
9.61_259.0217m/z	Glucose 6-P	-3.0937	1.2827	-2.8418	2.0393
8.92_195.0506m/z	Gluconic Acid	-3.0368	-0.8976	-3.3203	1.1769
2.62_391.2872m/z	Cdcholic Acid	-2.8201	0.3117	-0.2486	1.7529
8.64_202.0717m/z	N-AG	-2.8092	-3.0671	-1.3853	0.2989
6.12_218.1031m/z	Pantothenic Acid	-2.7786	-0.8496	-1.8857	2.7344
9.79_225.0987m/z	Carnosine	-2.7605	-2.6667	1.6559	-0.3467
9.91_866.1197m/z	Succinyl-CoA	-2.5792	2.5926	-1.6874	2.1156
7.84_282.0840m/z	Guanosine	-2.5228	-0.9369	2.1164	2.3972
1.13_227.2020m/z	Myristic Acid	-2.4457	-0.9996	-1.9200	-0.2242
10.07_191.0195m/z	Citric Acid	-2.4077	2.5009	-1.8783	2.0557
9.78_565.0474m/z	UDP-Glucose	-2.3618	2.5362	-1.8778	1.9924
10.27_505.9880m/z	ATP	-2.2992	3.2315	-2.9378	1.8833
11.22_481.9749m/z	CTP	-2.2313	1.4432	-4.1633	0.9752
10.57_408.0130m/z	dGTP	-2.1307	0.4344	-0.4031	2.0940
5.90_134.0472m/z	Adenine	-2.0599	-0.8917	2.9495	2.5346
9.39_214.0480m/z	G6P	-1.9979	-4.6907	0.9401	0.4390
8.81_202.0714m/z	N-A-Galactosamine	-1.9395	-2.0273	-2.9567	0.2263
10.14_341.1074m/z	Lactose	-1.9004	1.0859	0.4349	2.3785
9.34_131.0459m/z	L-Asparagine	-1.8536	-5.1755	-1.6721	0.4263

0.96_347.2240m/z	EPA	-1.8451	2.3910	-2.1641	1.6710
10.91_505.9879m/z	2'-DG-5'TP	-1.8000	3.1792	-3.7204	1.4472
2.16_164.0347m/z	4-Pyridoxic Acid	-1.7504	-0.5914	-2.3420	-0.1999
2.40_201.1139m/z	Sebaic Acid	-1.7251	-0.9473	-2.1713	0.9495
7.60_203.0825m/z	L-Tryptophan	-1.6111	0.8311	0.8294	3.6830
9.03_187.1084m/z	A-L-Lysine	-1.4245	1.7473	-0.2162	1.5543
9.43_362.0497m/z	GMP	-1.3585	-4.4900	-1.4912	1.2859
8.58_288.1197m/z	Ophthalmic Acid	-1.2045	-4.1109	-2.7970	2.1060
10.28_426.0207m/z	ADP	-0.7176	-1.3904	-4.9799	0.7970
5.88_245.0930m/z	N-A-Tryptophan	-0.7035	1.9192	-2.0047	1.7327
11.08_338.9880m/z	F1,5-BP	-0.6872	3.8318	-2.8391	1.7733
7.09_164.0717m/z	Phenylalanine	-0.6528	-1.1874	1.7160	3.7276
9.34_377.0850m/z	Trehalose	-0.6281	-4.8255	-3.4463	0.5169
4.15_187.0972m/z	Azelaic Acid	-0.5214	0.3813	2.4480	-1.1458
6.38_135.0313m/z	Hypoxanthine	-0.4524	0.4609	-1.6068	3.7447
9.19_145.0615m/z	L-Glutamine	-0.4521	-4.5690	0.1173	-1.3854
9.47_427.0068m/z	IDP	-0.2894	3.8749	-2.4989	-0.2607
11.12_220.9626m/z	3-PG Acid	-0.1875	-2.9709	-0.5048	0.0232
9.13_154.0622m/z	L-Histidine	-0.0249	-6.0384	1.1573	0.5838
9.46_711.2175m/z	Stachyose	-0.0240	-1.8737	4.0915	-0.9281
1.67_281.2484m/z	P Acid	-0.0208	-2.1281	5.9918	-0.1653
9.29_211.0002m/z	Ribose 5-P	0.0042	-5.3088	-2.5879	0.3701
8.90_387.1141m/z	Sucrose	0.1026	-0.6937	2.2820	-1.9968
8.90_342.1161m/z	Palatinose	0.1153	-0.7005	2.2784	-1.9063
0.73_283.2640m/z	Stearic Acid	0.1494	1.2457	-5.1839	-0.0002
10.71_442.0167m/z	GDP	0.2603	-0.4810	-4.0369	1.1835
1.66_255.2323m/z	Palmitic Acid	0.2962	-1.9058	5.9029	-0.1723
10.38_402.0097m/z	CDP	0.3384	-1.3166	-3.1832	1.1930
9.13_445.0526m/z	CDP-Ethanolamine	0.7761	-0.7203	1.1564	-1.0465
1.93_405.1906m/z	Cortisone	0.9021	-1.0666	1.7589	-0.6606
7.98_395.0540m/z	Rosmarinic Acid	1.2492	1.8026	0.8980	-1.1315
8.68_219.0508m/z	Shikimic Acid	1.3386	0.9999	2.5213	0.4058
5.79_173.0816m/z	Suberic Acid	1.3993	0.2825	1.7688	-2.2380
9.44_606.0738m/z	U-5'DP-AG	1.4194	-3.9288	-1.7994	1.7939
2.17_163.0407m/z	M-Vanillate	1.5245	1.9803	1.7358	1.7069
9.02_188.0563m/z	N-A-Glutamic Acid	1.5389	-1.4725	-4.3584	1.9646
8.85_540.0537m/z	ADP-Ribose	1.8988	-2.4933	-2.2450	2.1296
9.34_322.0443m/z	5'-CMP	1.9403	-5.5537	-2.0677	1.4755
8.71_237.0613m/z	Quinic Acid	1.9827	0.4635	2.2008	0.8706
11.17_579.0267m/z	UDP Glucuronic Acid	2.1310	-0.0766	-1.7162	1.6201
8.98_147.0527m/z	Glutamic Acid	2.2043	-3.0585	-1.6082	2.7583
5.75_156.0661m/z	N-Acetylproline	2.2271	0.7485	-0.8034	2.0453
9.19_347.0403m/z	IMP	2.6901	-3.8223	-1.9477	1.4659
7.28_260.0440m/z	cCMP	2.7840	3.3722	0.2125	-0.3016
9.03_171.0061m/z	G2-P	2.8322	-1.8263	-1.1515	2.4218

8.65_383.1135m/z	S-5'-Homocysteine	2.9137	-4.1650	-2.3720	1.1435
10.62_189.0880m/z	2,6-DH Acid	2.9424	-1.1604	-1.2257	0.5604
8.84_307.0826m/z	GSH reduced	2.9882	2.7260	-0.4841	1.4528
8.97_323.0285m/z	U-5MP	3.1789	-0.9290	1.0524	2.3436
8.48_808.1185m/z	Acetyl-CoA	3.1972	-1.8500	-4.0204	1.4139
5.99_312.0946m/z	Adenosine	3.3364	0.1368	0.9854	2.2246
6.33_243.0622m/z	Uridine	3.3701	-0.5588	1.0765	2.5532
7.01_266.0887m/z	2'-DG	3.4684	-0.2206	1.7232	2.1613
6.71_162.0418m/z	Pterin	3.4863	-2.7360	-1.2406	0.8604
8.94_766.1078m/z	CoA	3.5037	0.6781	-1.4685	1.1592
7.30_130.0872m/z	L-Isoleucine	3.5730	-1.1786	1.1065	2.1983
1.31_281.2483m/z	Oleic Acid	3.7936	1.2223	-3.0000	0.8277
1.28_253.2164m/z	Hexadecanoic Acid	3.9982	-0.8985	-2.3834	2.1396
8.56_347.0631m/z	AMP	4.3151	-2.8203	-1.0489	1.3063
8.45_167.0210m/z	Uric Acid	4.3918	-1.7981	-0.9647	1.8605
8.21_321.0492m/z	dTMP	4.4910	-1.9243	0.0850	1.7965
8.25_330.0604m/z	DAMP	4.5255	-2.0900	0.1587	1.4598
