# Supplementary figures and images for: Choosing an Optimal Sample Preparation in Caulobacter crescentus for Untargeted Metabolomics Approaches
Source: Metabolites. 2019 Sep 20;9(10):193. doi: 10.3390/metabo9100193 (PMC6836107; doi:10.3390/metabo9100193)

**A**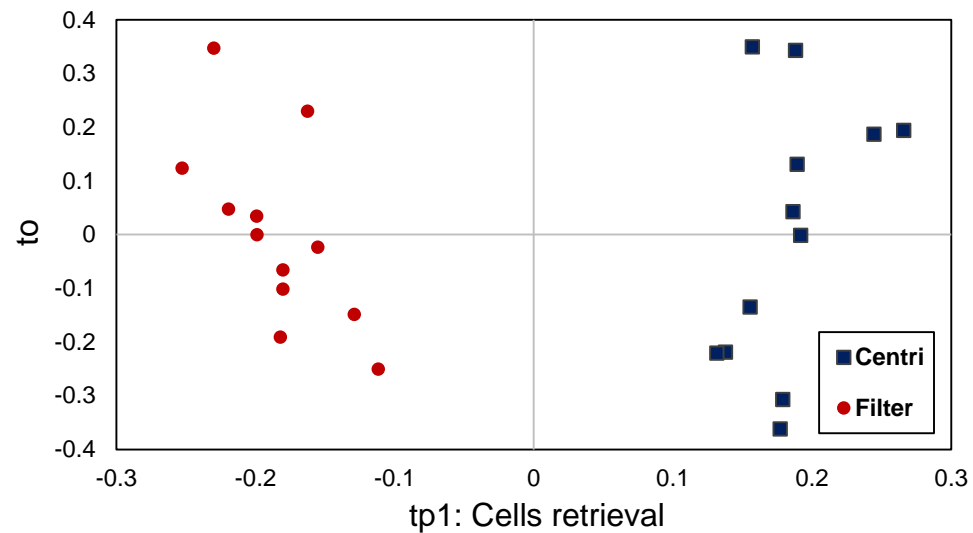**B**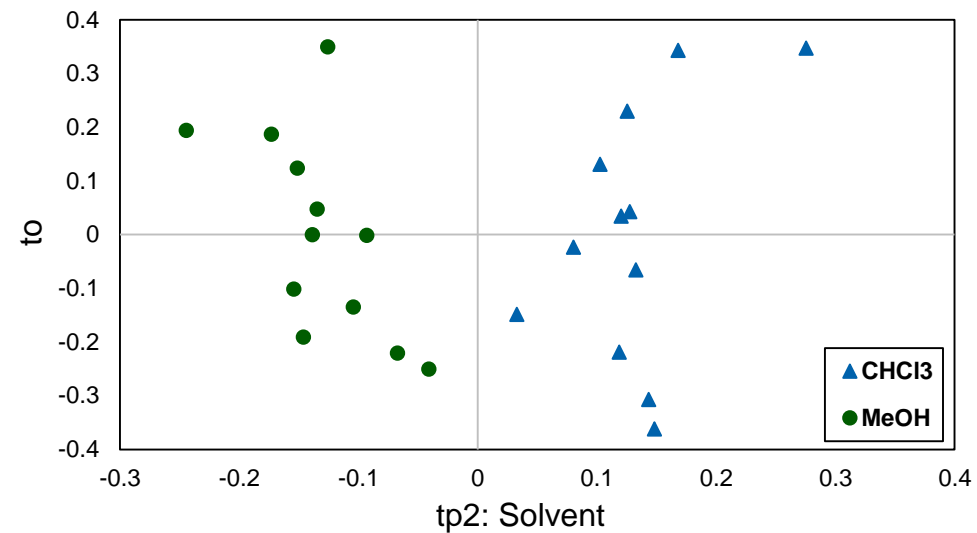**C**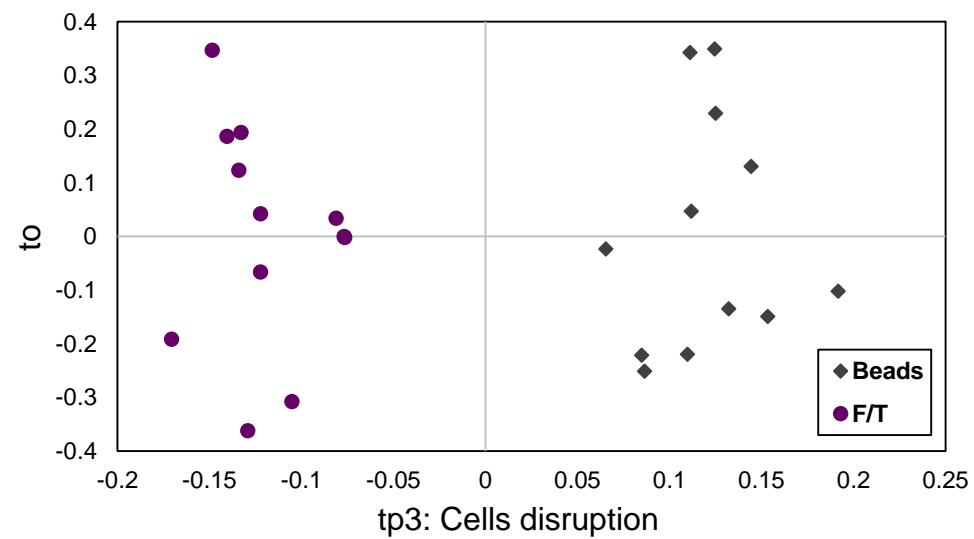

Supplement: Supplementary file 1 [file metabolites-09-00193-s001.zip › Supp Data/Figure S1.pdf]

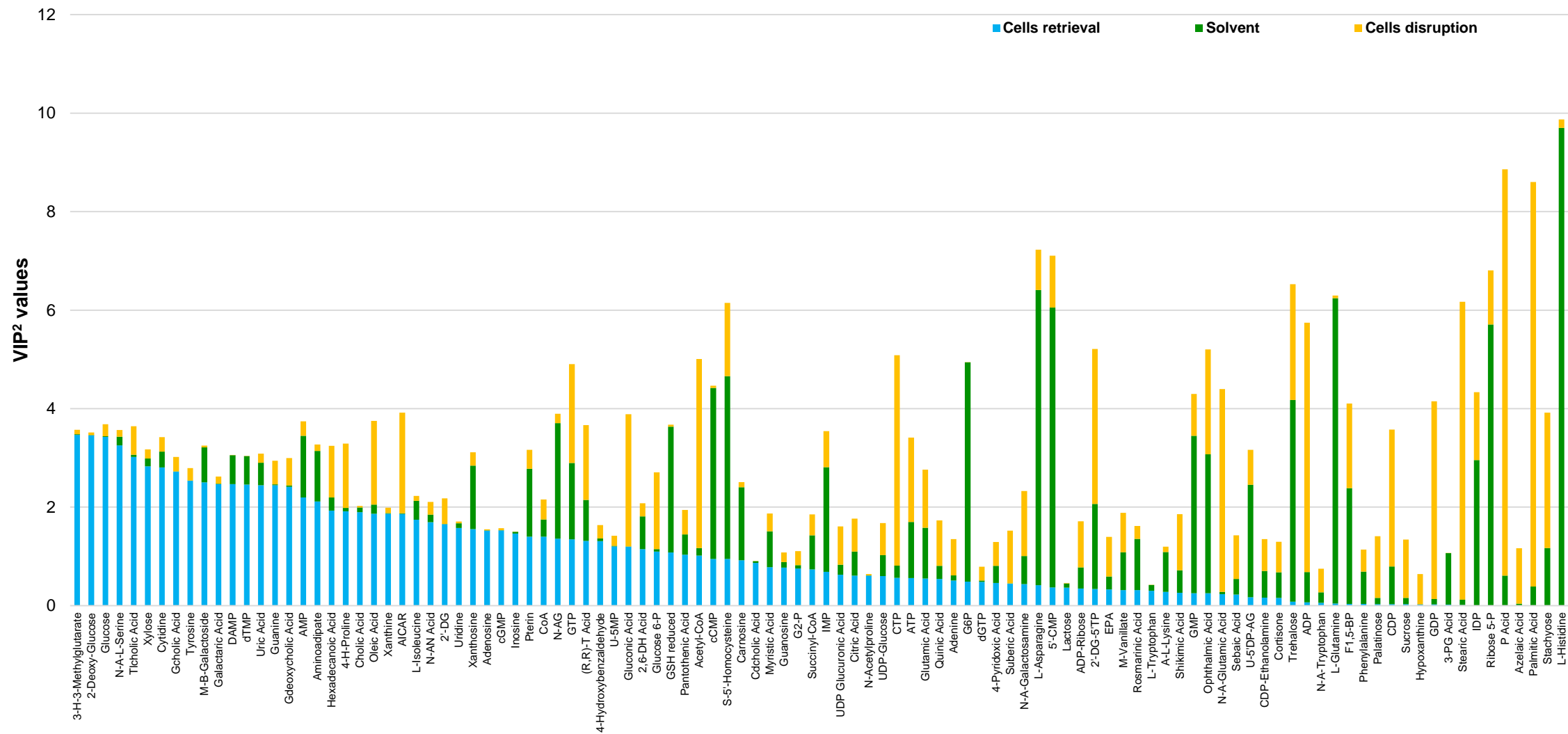

Supplement: Supplementary file 1 [file metabolites-09-00193-s001.zip › Supp Data/Figure S2.pdf]

A

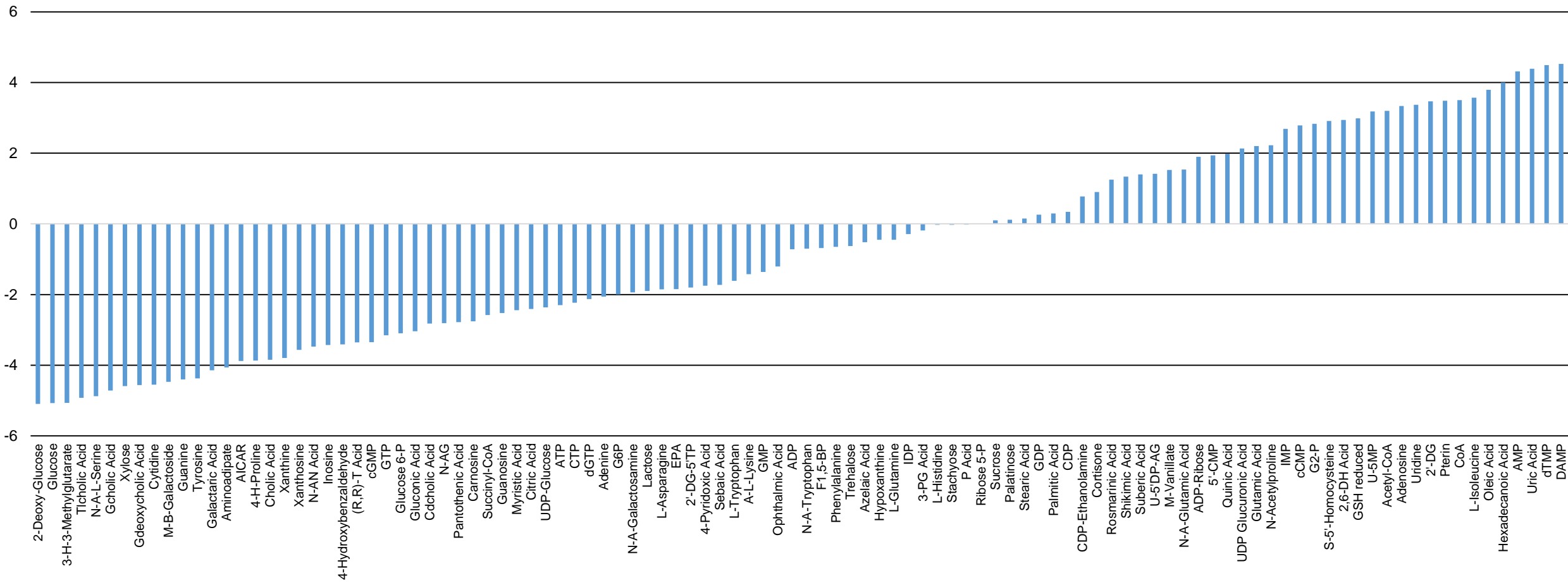

**B**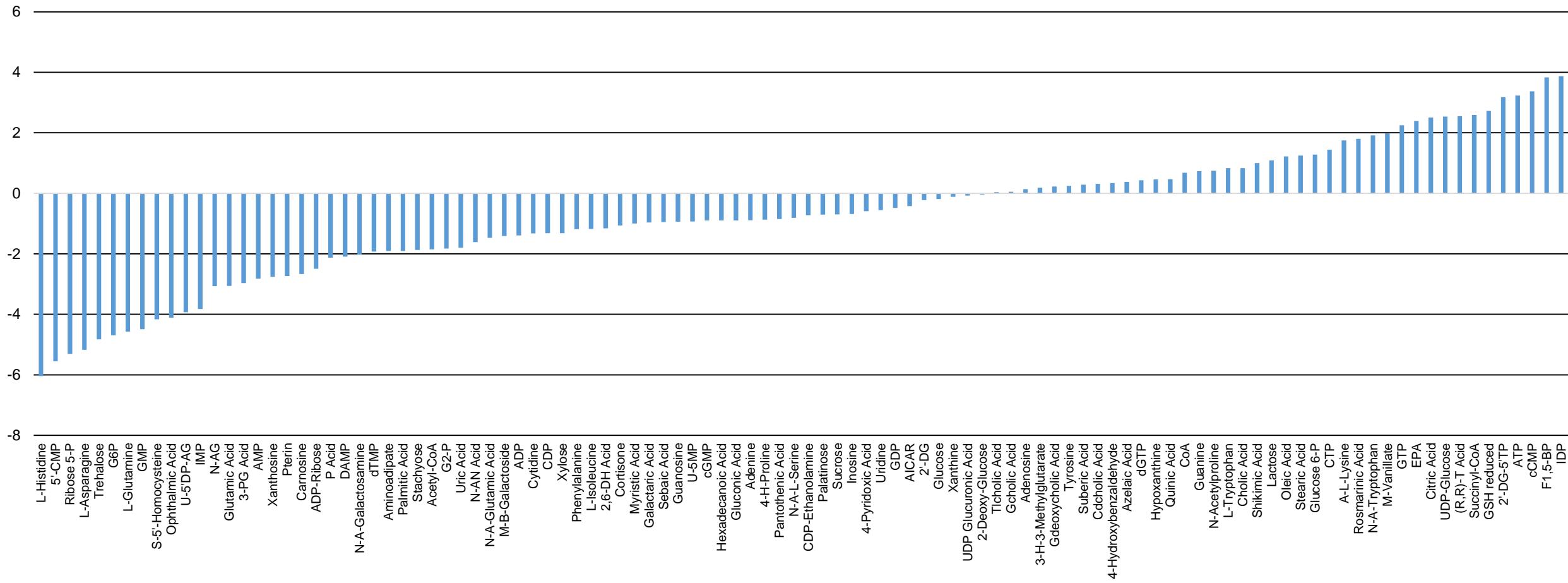

C

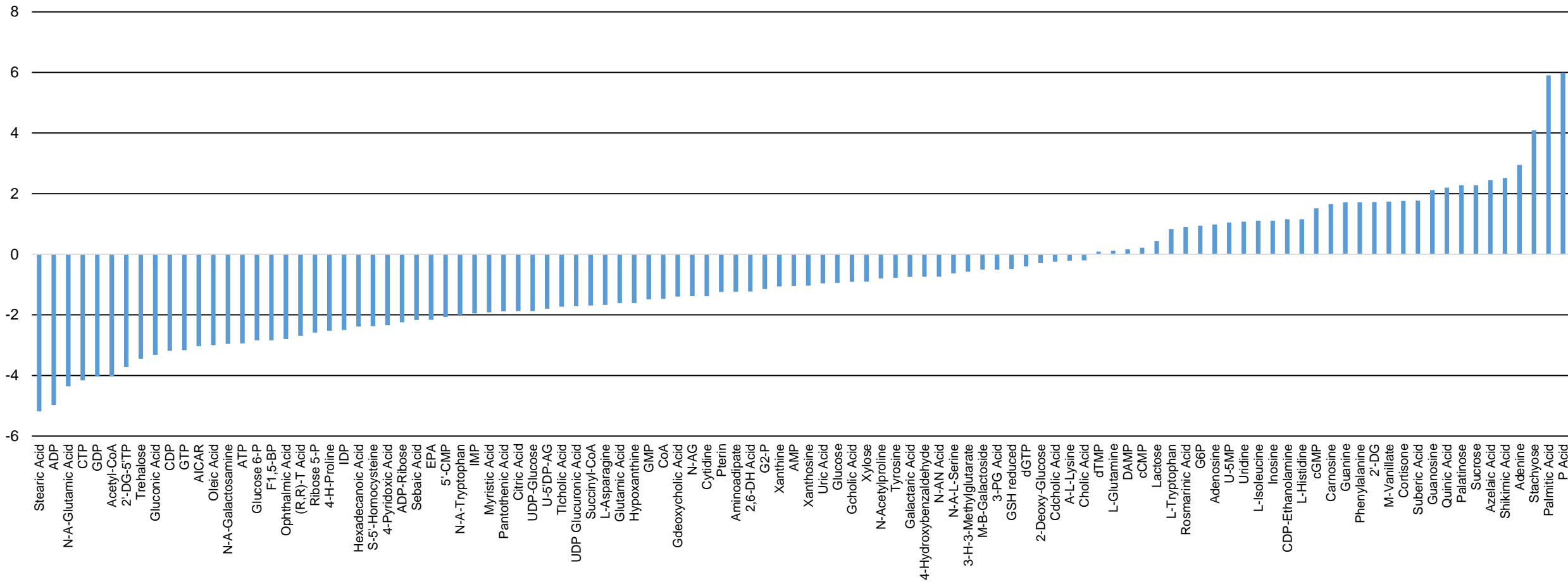

Supplement: Supplementary file 1 [file metabolites-09-00193-s001.zip › Supp Data/Figure S3.pdf]

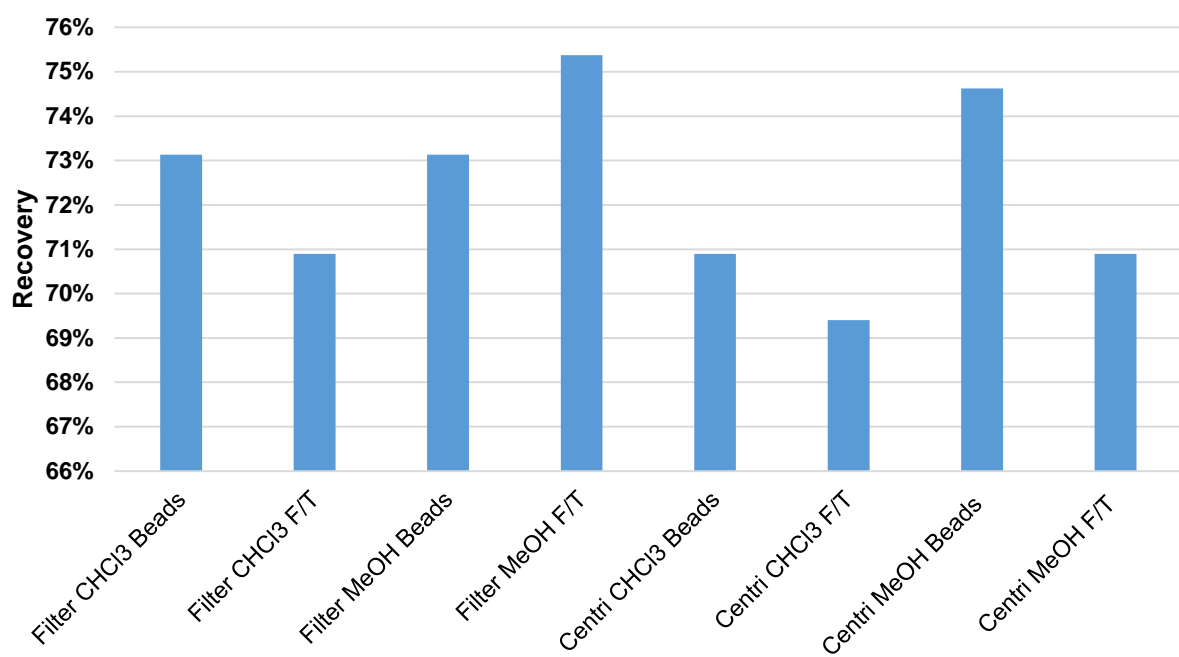

Supplement: Supplementary file 1 [file metabolites-09-00193-s001.zip › Supp Data/Figure S4.pdf]
